# A synthetic diphosphoinositol phosphate analogue of inositol trisphosphate†
†Electronic supplementary information (ESI) available: NMR spectra, additional DSF data and details of molecular docking experiments. See DOI: 10.1039/c8md00149a


**DOI:** 10.1039/c8md00149a

**Published:** 2018-06-04

**Authors:** Andrew M. Riley, Judith E. Unterlass, Vera Konieczny, Colin W. Taylor, Thomas Helleday, Barry V. L. Potter

**Affiliations:** a Medicinal Chemistry and Drug Discovery , Department of Pharmacology , University of Oxford , Mansfield Road , Oxford OX1 3QT , UK . Email: barry.potter@pharm.ox.ac.uk ; Fax: +44 (0)1865 271853 ; Tel: +44 (0)1865 271945; b Science for Life Laboratory , Department of Oncology-Pathology , Karolinska Institutet , SE-171 21 Solna , Sweden; c Department of Pharmacology , University of Cambridge , Tennis Court Road , Cambridge CB2 1PD , UK

## Abstract

Diphosphoinositol phosphates (PP-InsPs) are inositol phosphates (InsPs) that contain PP (diphosphate) groups. Converting a phosphate group in an InsP into a diphosphate has been reported to enhance affinity for some binding proteins. We synthesised 1-PP-Ins(4,5)P_2_, the first diphosphate analogue of the intracellular signalling molecule InsP_3_, and examined its effects on InsP_3_ receptors, which are intracellular Ca^2+^ channels. 1-PP-Ins(4,5)P_2_ was indistinguishable from InsP_3_ in its ability to bind to and activate type 1 InsP_3_ receptors, indicating that the diphosphate modification of InsP_3_ affected neither affinity nor efficacy. Nevertheless, 1-PP-Ins(4,5)P_2_ is the most potent 1-phosphate modified analogue of InsP_3_ yet identified. PP-InsPs are generally hydrolysed by diphosphoinositol polyphosphate phosphohydrolases (DIPPs), but 1-PP-Ins(4,5)P_2_ was not readily metabolised by human DIPPs. Differential scanning fluorimetry showed that 1-PP-Ins(4,5)P_2_ stabilises DIPP proteins, but to a lesser extent than naturally occurring substrates 1-PP-InsP_5_ and 5-PP-InsP_5_. The non-hydrolysable InsP_7_ analogues 1-PCP-InsP_5_ and 5-PCP-InsP_5_ showed comparable stabilising abilities to their natural counterparts and may therefore be promising substrate analogues for co-crystallisation with DIPPs.

## Abstract 2

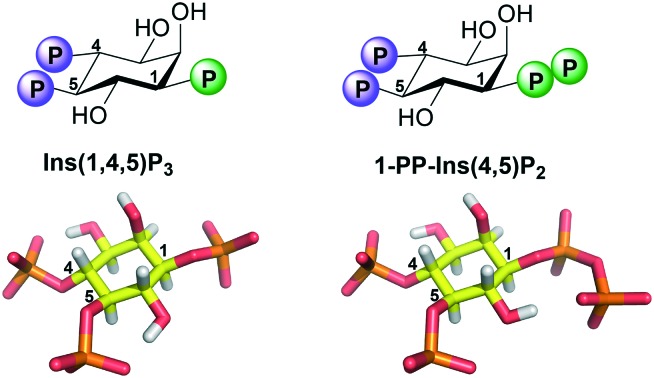
We describe the synthesis and biological evaluation of 1-PP-Ins(4,5)P_2_, the first diphosphate-containing analogue of the intracellular signalling molecule Ins(1,4,5)P_3_.

## Introduction

**Fig. 1:**
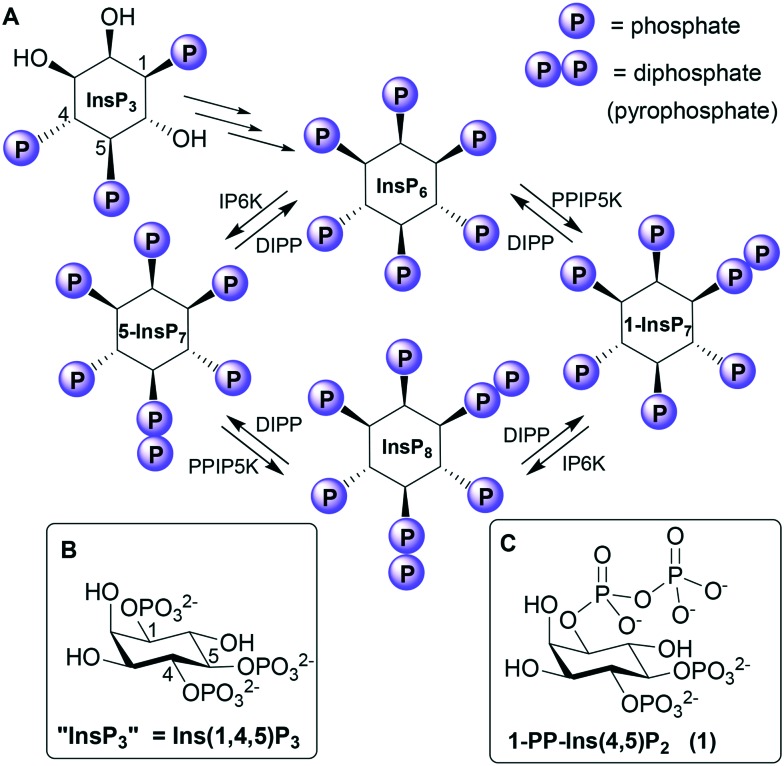
A. Biosynthetic pathway connecting Ins(1,4,5)P_3_ to the PP-InsPs. IP6K, inositol hexakisphosphate 5-kinase; PPIP5K, diphosphoinositol pentakisphosphate kinase; DIPP, diphosphoinositol polyphosphate phosphohydrolase. “InsP_3_”, “5-InsP_7_”, “1-InsP_7_” and “InsP_8_” are alternative names for Ins(1,4,5)P_3_, 5-PP-InsP_5_, 1-PP-InsP_5_ and 1,5-[PP]_2_-InsP_4_, respectively. B. Structure of Ins(1,4,5)P_3_. C. Structure of the synthetic analogue 1-PP-Ins(4,5)P_2_ (**1**).

The *myo*-inositol phosphates (InsPs) are a family of intracellular signalling molecules containing monophosphate (P) and diphosphate (PP) groups arranged around the hexahydroxycyclohexane ring of *myo*-inositol (Ins).^1^ InsPs regulate many cellular processes, the best known being the release of Ca^2+^ from intracellular stores by d-*myo*-inositol 1,4,5-trisphosphate (InsP_3_), which binds to receptors on the endoplasmic reticulum.^2^ InsP_3_ is converted *via* a series of enzymatic phosphorylations^3^ into InsP_6_ (Fig. 1), which can then be further phosphorylated to give highly charged PP-InsPs containing diphosphate (pyrophosphate) groups.^4^,^5^

InsP_3_ receptors (IP_3_Rs) are tetrameric intracellular Ca^2+^ channels, expressed in most animal cells.^2^ When InsP_3_ binds to the *N*-terminal InsP_3_-binding core (IBC) of all four IP_3_R subunits,^6^ conformational changes propagate to the central pore. The pore then opens, allowing Ca^2+^ to flow into the cytosol, where it regulates many intracellular processes. The vicinal 4,5-bisphosphate structure of InsP_3_ is crucial (if not absolutely essential^7^) for activating IP_3_Rs because it cross-links the two domains of the clam-like IBC, pulling them together and initiating the conformational changes. The 1-phosphate group has a less direct, but enhancing, effect on activity.^8^

Although PP-InsP signalling is thought to be more evolutionarily ancient than InsP_3_-mediated mobilisation of Ca^2+^,^9^ much less is known about the functions and protein targets of PP-InsPs. Nevertheless, evidence is accumulating that PP-InsPs play important roles at the interface of cell signalling and metabolism in the regulation of bioenergetic and phosphate homeostasis.^4^,^5^ Possible receptors for PP-InsPs include the PH (pleckstrin homology) domains^10^,^11^ and SPX (SYG1/Pho81/XPR1) domains^12^,^13^ of proteins. PP-InsPs may also exert some of their effects by direct non-enzymatic diphosphorylation of target proteins.^14^

Phosphorylating a phosphate monoester in an InsP_*n*_ to give a PP-InsP_*n*–1_ not only increases the overall negative charge of the molecule, but also changes its shape, solvation and metal complexation properties. Unsurprisingly, therefore, a diphosphate group may alter ligand affinity for protein binding sites.^4^ For example, some PH domains that bind InsP_6_ bind 5-InsP_7_ with higher affinity,^10^,^11^ while 1-InsP_7_ and InsP_8_ are weaker.^11^ In contrast, both 1-InsP_7_ and 5-InsP_7_ stimulate synthesis of inorganic polyphosphate (polyP) by the vacuolar transporter chaperone (VTC) by binding to its SPX domain, while InsP_6_ is inactive and InsP_8_ is 20-fold more potent.^13^ PP-InsPs can be dephosphorylated back to InsPs by diphosphoinositol polyphosphate phosphohydrolases (DIPPs, Fig. 1), which specifically hydrolyse the diphosphate group, leaving a phosphate monoester and liberating inorganic phosphate.^3^,^15^

Given that introducing a diphosphate into an InsP may modify its interaction with proteins, we were interested in the possible effects of converting one of the phosphate groups in InsP_3_ into a diphosphate. The 1-phosphate group of InsP_3_ has been a popular target for synthetic elaboration of InsP_3_ since early structure–activity studies showed that it is much more tolerant of modification than the 4- or 5-phosphate groups.^8^ Interest in the role of the 1-phosphate group was further stimulated by the discovery in 1993 of the adenophostins, fungal metabolites that are highly potent InsP_3_ receptor ligands.^16^ The adenophostins contain a glucopyranoside 3,4-bisphosphate structure that mimics the *myo*-inositol 4,5-bisphosphate of InsP_3_ but intriguingly, their third phosphate group is located on a separate (ribofuranoside) ring, suggesting that repositioning this phosphate group may enhance affinity.^17^

The X-ray structure^18^ of the IBC of type 1 InsP_3_ receptor bound to InsP_3_ confirmed the area of the binding pocket around the 1-phosphate of bound InsP_3_ to be relatively open. Our molecular docking experiments using this structure suggested that a 1-diphosphate should bind well to this region. We therefore set out to synthesise the 1-diphosphate analogue of InsP_3_, *i.e.* 1d-diphospho-*myo*-inositol 4,5-bisphosphate [1-PP-Ins(4,5)P_2_ (**1**), Fig. 1] and examine its interaction with InsP_3_ receptors.

We were also interested to examine the interaction of 1-PP-Ins(4,5)P_2_ with DIPPs. Although DIPPs can hydrolyse the PP groups of highly phosphorylated PP-InsPs (Fig. 1), inorganic polyphosphate, 5-phosphoribosyl 1-pyrophosphate and nucleotide dimers,^3^,^15^ their catalytic mechanisms are poorly understood. 1-PP-Ins(4,5)P_2_ contains the target 1-PP structure found in the known DIPP substrate 1-PP-InsP_5_, but presented in the context of a molecule with only two phosphate monoester groups. There are no reports in the literature on whether “lower” PP-InsPs such as 1-PP-Ins(4,5)P_2_ could be recognised by the active sites of DIPPs.

## Results and discussion

### Chemistry

**Scheme 1:**
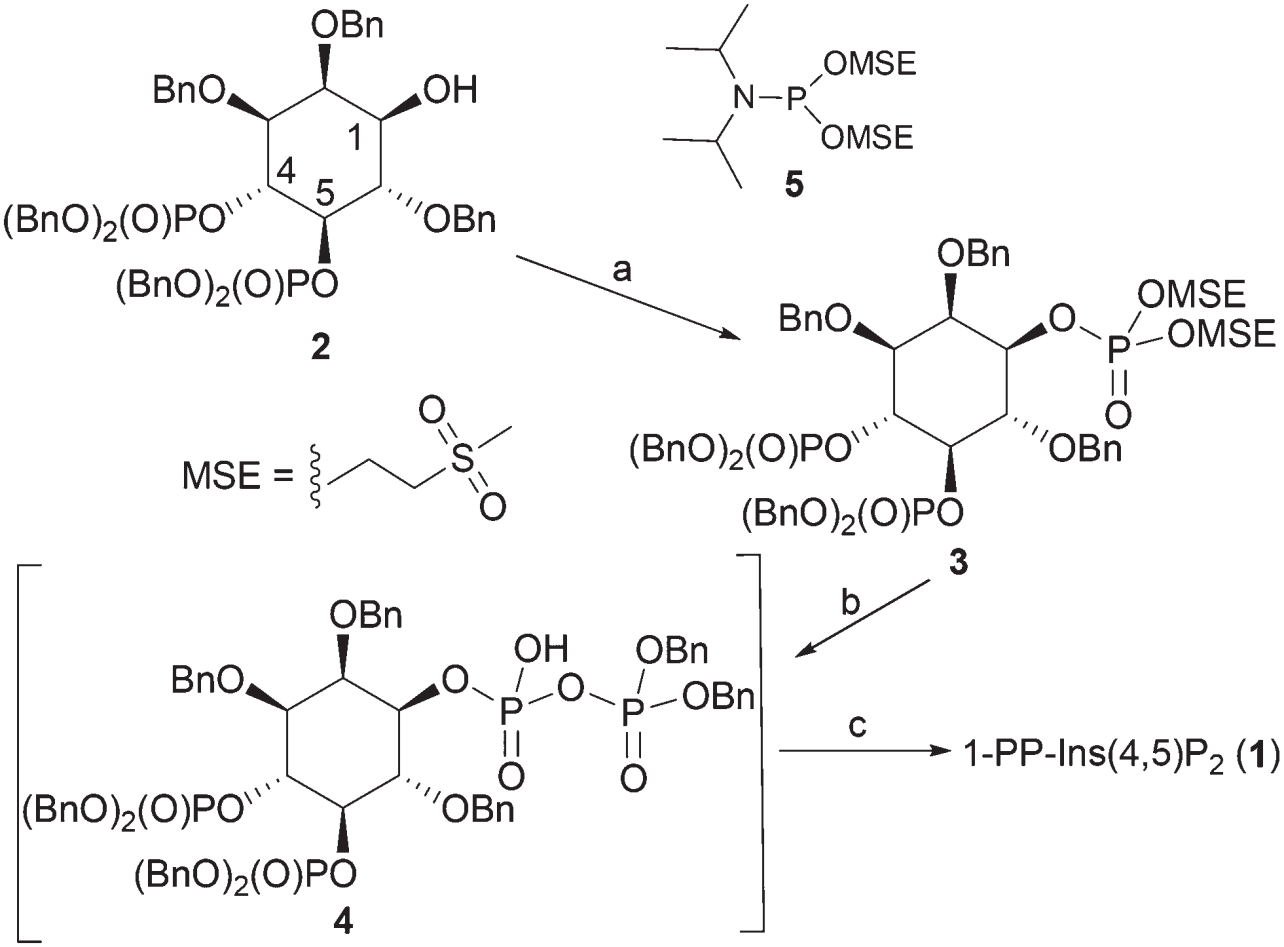
Synthesis of 1-PP-Ins(4,5)P_2_ (**1**). Reagents and conditions: a. i. 5-phenyl-1*H*-tetrazole, **5**, CH_2_Cl_2_; ii. *m*CPBA, CH_2_Cl_2_, 89%; b. i. DBU, BSTFA, CDCl_3_; ii. MeOH, then TFA; iii. 5-phenyl-1*H*-tetrazole, (BnO)_2_PNPr^i^_2_, CH_2_Cl_2_; iv. *m*CPBA, CH_2_Cl_2_, 90%; c. i. H_2_, Pd(OH)_2_/C, MeOH, H_2_O, NaHCO_3_; ii. Ion-exchange chromatography on Q-Sepharose Fast Flow resin, 57%. Bn, benzyl.

The synthesis of 1-PP-Ins(4,5)P_2_ (**1**) begins from the known alcohol **2** (ref. 19 and 20) (Scheme 1). To construct the diphosphate unit at *O*-1, we employed a modification of a recently described strategy,^21^,^22^ in which a temporarily protected phosphate group is introduced and then selectively deprotected to reveal a phosphate monoester. This phosphate is then phosphitylated to give a mixed P(iii)–P(v) anhydride, which is oxidised to a partially protected pyrophosphate unit. Removal of all protecting groups by catalytic hydrogenolysis then yields the target PP-InsP. We reasoned that it might be possible to employ methylsulfonylethyl (MSE)^23^,^24^ as a temporary phosphate protecting group in this sequence. The MSE group can be removed by β-elimination, similar to the better-known β-cyanoethyl (β-CE)^22^,^25^ group. However, the MSE group is unaffected by catalytic hydrogenation, affording greater synthetic versatility, and the required phosphitylating reagent, phosphoramidite **5**, is a stable crystalline solid.

Thus, the 1-OH group in **2** was reacted with **5** in the presence of 5-phenyl-1*H*-tetrazole to give an intermediate MSE-protected phosphite triester. Oxidation using *m*CPBA then gave **3**, containing the MSE-protected phosphate triester at *O*-1. The diphosphate unit at *O*-1 was then constructed using a sequence of transformations carried out as described previously,^21^,^22^,^25^ with slight modifications. The progress of each step was carefully monitored by ^31^P NMR spectroscopy (see Experimental section and ESI†). The protected diphosphate **4** was found to be rather unstable and was immediately deprotected by catalytic hydrogenolysis at atmospheric pressure. A final purification step by gradient elution anion exchange chromatography on Q-Sepharose Fast Flow resin gave 1-PP-Ins(4,5)P_2_ (**1**) as the triethylammonium salt, which was accurately quantified by total phosphate assay.

### Interactions of 1-PP-Ins(4,5)P_2_ with type 1 InsP_3_ receptors

**Fig. 2:**
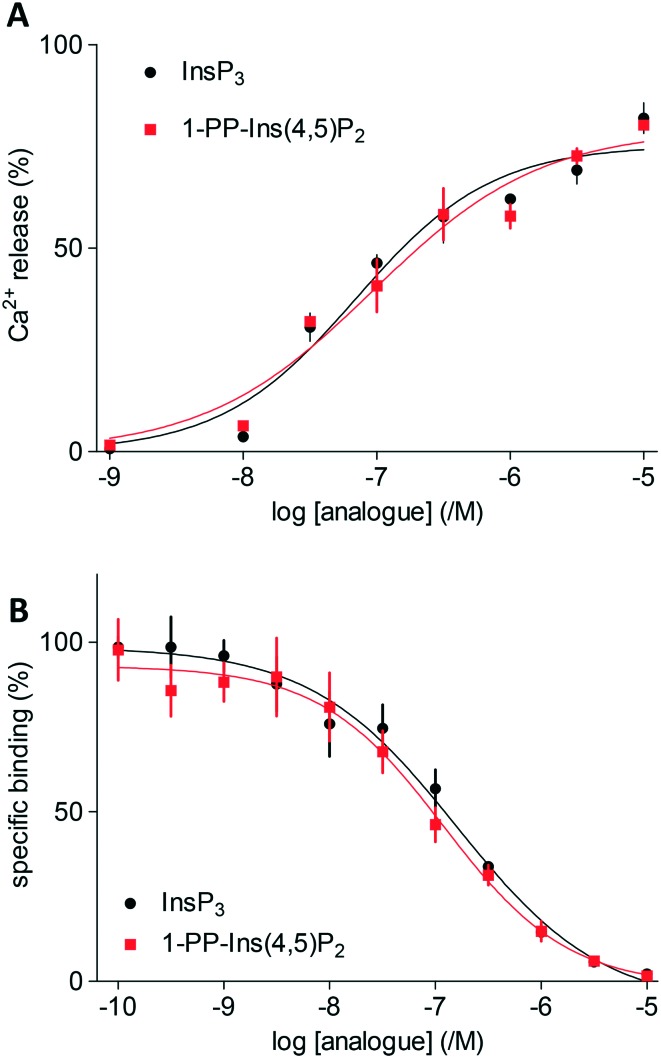
A. Ca^2+^ release from intracellular stores of DT40 cells expressing type 1 InsP_3_ receptors stimulated by InsP_3_ and 1-PP-Ins(4,5)P_2_ (**1**). Results are shown as % of Ca^2+^ content of intracellular stores. B. Equilibrium competition binding with ^3^H-InsP_3_ and InsP_3_ or 1-PP-Ins(4,5)P_2_ (**1**) using membranes from Sf9-IP_3_R1 cells in CLM containing 1.5 mM MgATP. Results are means ± s.e.m., *n* = 3.

Both InsP_3_ and 1-PP-Ins(4,5)P_2_ (**1**) stimulated a concentration-dependent release of Ca^2+^ from the intracellular stores of permeabilised DT40 cells expressing type 1 InsP_3_ receptors (Fig. 2A). The maximal Ca^2+^ release evoked by each ligand and the half-maximally effective concentration (EC_50_) were similar for **1** and InsP_3_ (Fig. 2A). Membranes from Sf9 cells expressing rat type 1 InsP_3_ receptors were used for equilibrium competition binding studies with ^3^H-InsP_3_, because these membranes express full-length type 1 InsP_3_ receptors at ∼20-fold higher levels than cerebellar membranes, the richest endogenous source. The experiments were carried out in cytosol-like medium (CLM, pH 7.3) containing 1.5 mM Mg-ATP to match the conditions used for Ca^2+^-release assays.

In agreement with the Ca^2+^-release assays, 1-PP-Ins(4,5)P_2_ (**1**) bound with the same affinity as InsP_3_ to InsP_3_ receptors (Fig. 2B). Thus, the two compounds were essentially indistinguishable in both functional and binding assays (Table 1). Rapid chemical hydrolysis of **1** could in principle explain the similar behaviour of InsP_3_ and **1**, but we saw no evidence that **1** is unstable. The ^31^P NMR spectrum of **1** in D_2_O (see ESI†) was unchanged after the sample solution had been kept for several days at room temperature, followed by one year at 4 °C.

**Table 1:** Interactions of InsP_3_ and 1-PP-Ins(4,5)P_2_ (**1**) with type 1 InsP_3_ receptors (*n* = 3)

	Ca^2+^ release			Binding^*a*^	
	pEC_50_/M	EC_50_/nM	% release	p*K*_d_/M	*K*_d_/nM
InsP_3_	7.21 ± 0.08	62	82 ± 4	6.89 ± 0.07	128
1-PP-Ins(4,5)P_2_ (**1**)	7.17 ± 0.11	68	80 ± 1	6.96 ± 0.05	110

^*a*^Binding was done using Sf9 cell membranes overexpressing rat type 1 InsP_3_ receptors in CLM (pH 7.3) containing 1.5 mM Mg-ATP to match the conditions used in the Ca^2+^ release assay.

**Fig. 3:**
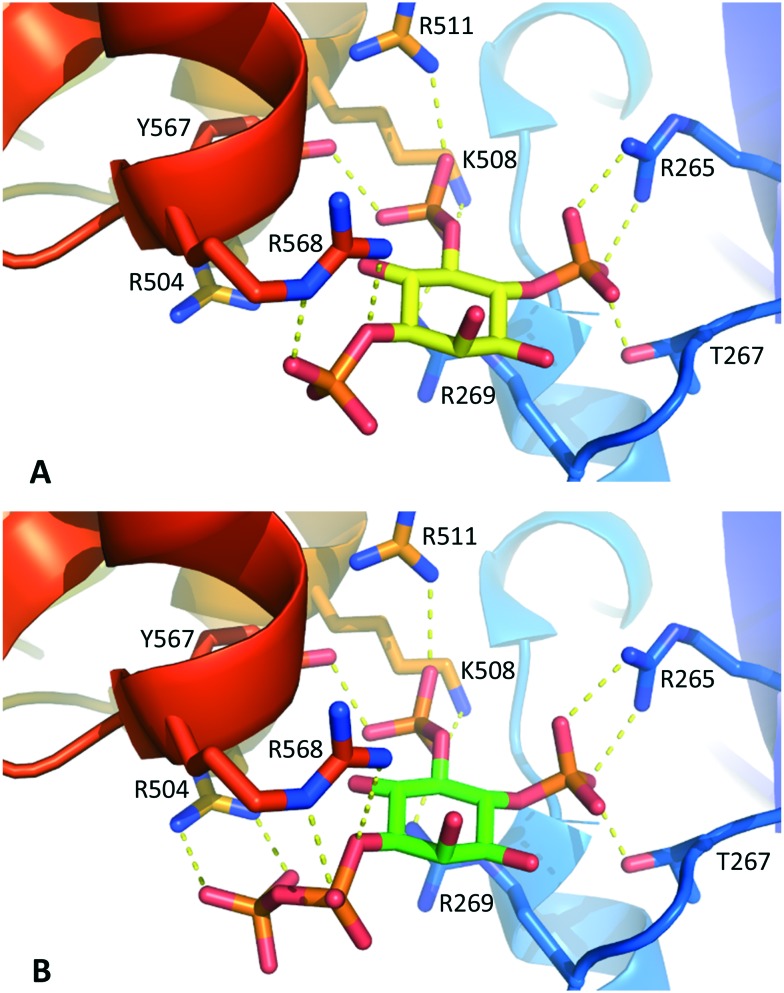
A. Interactions of InsP_3_ with the IBC of type 1 InsP_3_ receptors, based on the X-ray crystal structure of IP_3_R1 in complex with InsP_3_ (ref. 18) (1N4K). B. Model of 1-PP-Ins(4,5)P_2_ (**1**) in the IBC produced by molecular docking (see Experimental section and ESI† for details). For clarity, water molecules are not shown.

Molecular docking experiments (see Experimental section and ESI† for details) using the X-ray crystal structure of the IBC of type 1 InsP_3_ receptor^18^ suggested that the diphosphate group in **1** should be well-tolerated by the InsP_3_-binding pocket and may be capable of forming additional hydrogen bonds with residues in the binding site (Fig. 3). Nevertheless, it is well known that attempts to optimise drug candidates by adding polar groups may fail because the expected enthalpic gains from new polar interactions are opposed by ligand desolvation penalties and unfavourable entropic effects, resulting in no gain in binding affinity.^26^ Such compensatory effects may underlie the similar affinities of **1** and InsP_3_ for type 1 InsP_3_ receptors.

### Interaction of 1-PP-Ins(4,5)P_2_ with DIPPs

The dephosphorylation of PP-InsPs is catalysed by diphosphoinositol polyphosphate phosphohydrolases (DIPPs), which selectively cleave the diphosphate (PP) to give a phosphate monoester and inorganic phosphate (Pi).^3^ Humans express four DIPP types: DIPP-1 is the product of the NUDT3 gene; DIPP-2 (of which there are two isoforms, DIPP-2α and DIPP-2β, produced by alternative splicing) is the product of NUDT4; DIPP-3α is the product of NUDT10 and DIPP-3β is the product of NUDT11.^3^ We examined the interaction of 1-PP-Ins(4,5)P_2_ (**1**) with all four DIPPs in comparison with two naturally-occurring substrates 1-PP-InsP_5_ and 5-PP-InsP_5_ (“1-InsP_7_” and “5-InsP_7_”, respectively) and also with the alternative substrates diadenosine polyphosphates Ap_3_A and Ap_5_A. Non-hydrolysable InsP_7_ analogues 1-PCP-InsP_5_ (ref. 27) and 5-PCP-InsP_5_ (ref. 28) were independently synthesised and included as controls.

**Fig. 4:**
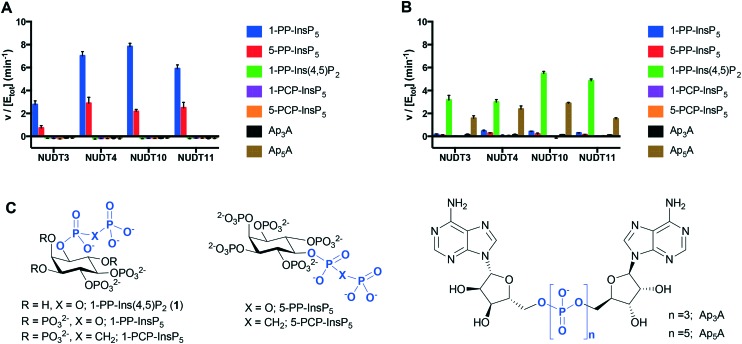
Specific activities of DIPPs with 1-PP-Ins(4,5)P_2_ (**1**), known substrates 1-PP-InsP_5_ and 5-PP-InsP_5_ and controls. Experiments were conducted in buffer containing Mg^2+^ (A) or Mn^2+^ (B). Data shown represent the formed concentration of Pi (micromolar) per enzyme concentration (micromolar) per minute. A630 was converted to Pi concentration (micromolar) using the equation A630 = 0.01897[P_i_] – 0.5877 (Mg^2+^ containing buffer) or A630 = 0.01923[P_i_] + 0.1053 (Mn^2+^ containing buffer). C. Structures of compounds examined, including methylenebisphosphonate (PCP) analogues of InsP_7_, and diadenosine polyphosphates Ap_3_A and Ap_5_A.

With Mg^2+^ present in the buffer, 1-PP-InsP_5_ and 5-PP-InsP_5_ were rapidly metabolised by all four DIPPs (Fig. 4A). The rate of hydrolysis of 1-PP-InsP_5_ was significantly higher than that for 5-PP-InsP_5_ in each case. This finding is in agreement with a previous study.^15^ As expected, the PCP analogues were not metabolised, confirming that DIPPs can hydrolyse only the diphosphate unit and not the phosphate monoesters. Ap_3_A and Ap_5_A were unaffected by all four enzymes in Mg^2+^-containing buffer, an observation that had been reported for NUDT10 and NUDT11, but not for NUDT3 and NUDT4.^29^ Perhaps surprisingly, 1-PP-Ins(4,5)P_2_ (**1**) was also not metabolised under these conditions. The presence of a divalent cation is required for the activity of NUDT10 and NUDT11 and also for NUDT3.^3^ When Mg^2+^ in the buffer was replaced by Mn^2+^, **1** was now hydrolysed by the DIPPs, while 1-PP-InsP_5_ and 5-PP-InsP_5_ resisted hydrolysis. In addition, Ap_5_A now also behaved as a substrate for all four DIPPs (Fig. 4B). In the absence of enzyme none of the compounds, including **1**, showed any sign of hydrolysis during the time course of the experiment in the presence of either Mg^2+^ or Mn^2+^-containing buffers. This further supports our conclusion above that **1** was not hydrolysed to InsP_3_ during the InsP_3_ receptor assays.

**Fig. 5:**
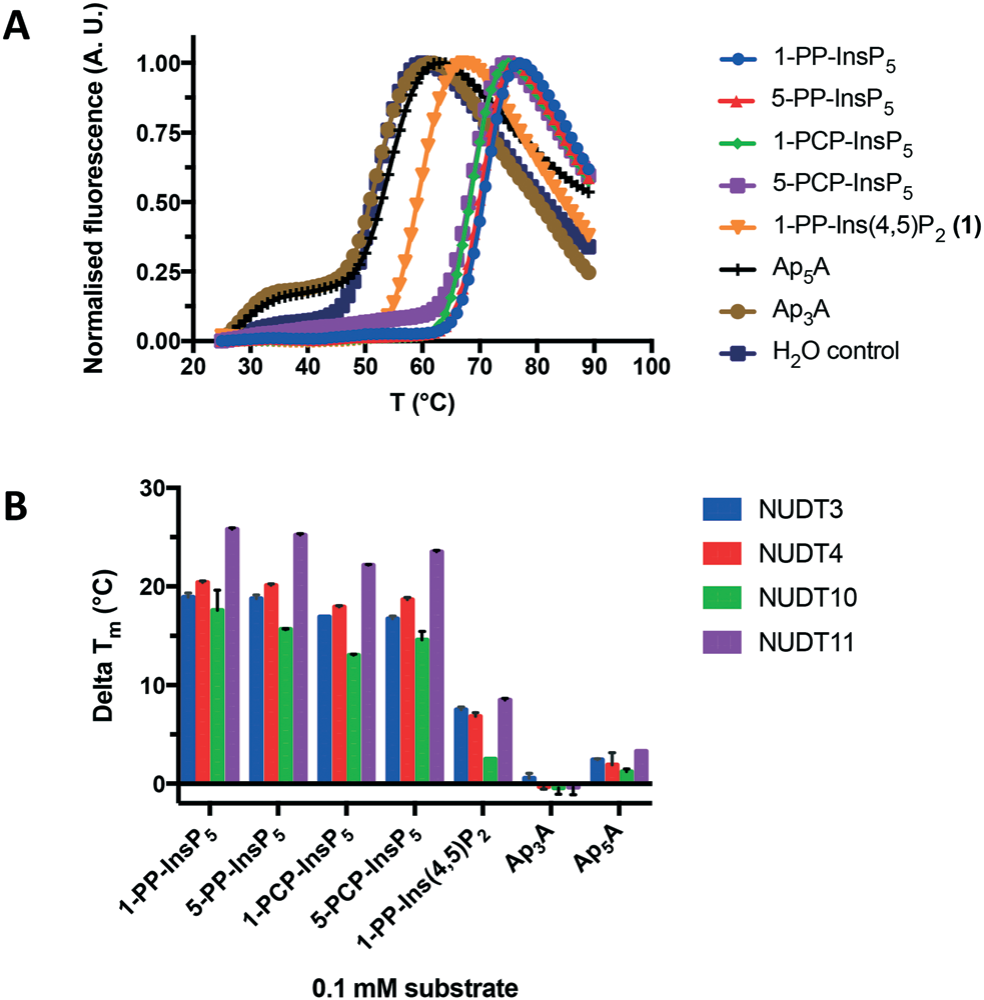
A. Effect of 1-PP-Ins(4,5)P_2_ (**1**) and other compounds shown in Fig. 4C on the melting temperature (*T*_m_) of NUDT3, measured using differential scanning fluorimetry (DSF). B. Comparison of melting temperature shifts (delta *T*_m_) induced by all compounds for all four DIPPs examined.

Next, we used differential scanning fluorimetry (DSF) to measure the ability of the compounds to stabilise NUDT3 (DIPP1). While the effects of Ap_3_A and Ap_5_A were not significantly different from control (Fig. 5A), 1-PP-Ins(4,5)P_2_ (**1**) raised the melting temperature (*T*_m_) of NUDT3 by approx. 5 °C at a concentration of 0.1 mM. As expected, the more highly phosphorylated 1-PP-InsP_5_ had much stronger effects, resulting in a *T*_m_-shift of 20–25 °C. Similar DSF experiments were then carried out for NUDT4, NUDT10 and NUDT11. Ap_3_A did not stabilise any of the DIPPs, which supports our results for the activity assay. The results are summarised in Fig. 5B.

**Fig. 6:**
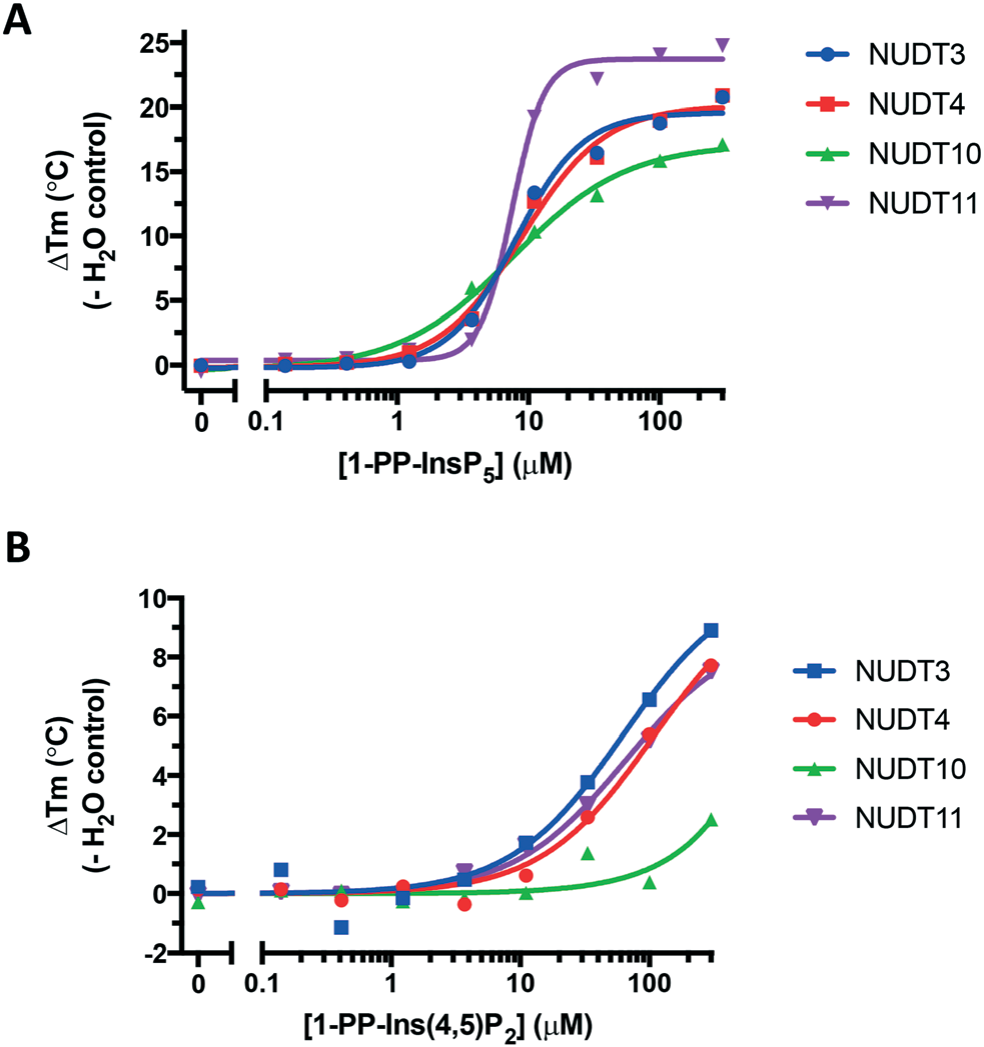
Dose–response curves showing stabilisation of all four DIPPs by A. 1-PP-InsP_5_ and B. 1-PP-Ins(4,5)P_2_ (**1**). Note the different *y*-axis scales in A and B.

We obtained further DSF data over a range of ligand concentrations for 1-PP-InsP_5_ and 1-PP-Ins(4,5)P_2_ (**1**), constructing dose–response curves for the two compounds (Fig. 6). It is interesting to note that the effect of **1** on NUDT10 was significantly lower compared to the other DIPPs and especially compared to NUDT11 (Fig. 6B). NUDT10 and NUDT11 have identical protein sequences apart from residue 89, which is either proline (NUDT10) or arginine (NUDT11).

Noting the strong stabilisation of all the proteins by the PCP analogues, we obtained further DSF data over a range of ligand concentrations for 1-PCP-InsP_5_ and 5-PCP-InsP_5_ (ESI† Fig. S4 and S5) and calculated *K*_D_ values from these curves (ESI† Tables S1 and S2). We found that, in some cases, the PCP analogues had binding affinities comparable to those of their natural PP-containing ligands.

## Conclusions

Replacing a phosphate group in an inositol phosphate ligand with a diphosphate (PP) group can modify the interaction of the ligand with target proteins.^10^–^13^ Structure–activity studies have previously shown that the 1-phosphate group of InsP_3_ is amenable to synthetic modification, and molecular docking experiments suggested that a 1-diphosphate group should be well-tolerated by the binding site of the InsP_3_ receptor. We therefore synthesised 1-PP-Ins(4,5)P_2_ (**1**), the first PP-containing analogue of InsP_3_. Using assays of Ca^2+^-release through type 1 InsP_3_ receptors, we found that **1** was equipotent to InsP_3_ and in binding assays its affinity was indistinguishable from that of InsP_3_. Thus, the 1-diphosphate modification of InsP_3_ does not affect its affinity for or activity at type 1 InsP_3_ receptors. Nevertheless, **1** is the first Ca^2+^-releasing PP-InsP and also the most potent P-1 modified ligand of InsP_3_ receptors yet identified.‡
‡A synthetic InsP_3_ derivative featuring 4-carboxy-malachite green conjugated to the 1-phosphate group was reported to have ∼170-fold higher affinity than InsP_3_ for an *N*-terminal fragment of type 1 InsP_3_ receptors.^43^ In our hands, this compound was ∼5-fold less potent than InsP_3_ at each InsP_3_ receptor subtype and had an affinity ∼7-fold less than InsP_3_ for type 1 InsP_3_ receptors.^44^


The novel diphosphate compound **1** was not metabolised by DIPPs in the presence of Mg^2+^-containing buffer, while the naturally-occurring InsP_7_ isomers, 5-PP-InsP_5_ and 1-PP-InsP_5_ were rapidly hydrolysed. Conversely, in the presence of Mn^2+^, **1** was hydrolysed while the two InsP_7_ isomers were unaffected. Synthetic PCP-containing analogues of the InsP_7_s were not hydrolysed under any conditions examined, but when evaluated for their ability to stabilise DIPP proteins using differential scanning fluorimetry (DSF), they gave temperature shifts comparable to their natural PP-containing equivalents. This strongly suggests that 1-PCP-InsP_5_ and 5-PCP-InsP_5_ could be promising ligands for co-crystallisation studies with DIPPs.

Could 1-PP-Ins(4,5)P_2_ be an endogenous molecule? The mammalian enzymes known to synthesise PP-InsPs are 5-diphosphoinositol pentakisphosphate kinases (PPIP5Ks) and inositol hexakisphosphate kinases (IP6Ks). Inositol phosphate multikinase (IPMK) has also been reported to synthesise PP-InsP_4_ from InsP_5_*in vitro*,^30^ but the products of InsP_3_ phosphorylation by IPMK are Ins(1,3,4,5)P_4_ and/or Ins(1,4,5,6)P_4_.^31^ Phosphorylation of lower InsPs by PPIP5Ks seems unlikely, considering the constraints of the catalytic site^32^ and the recently discovered capture site;^22^ even Ins(1,3,4,5,6)P_5_ is not phosphorylated.^32^ Recombinant Kcs1p, a yeast homologue of IP6K1, was reported to phosphorylate InsP_3_ slowly, although the identities of the products could not be determined.^33^ Later work confirmed that InsP_3_ was phosphorylated by Kcs1 and the product was identified as Ins(1,3,4,5)P_4_ (*i.e.* in this case, Kcs1 functioned as a 3-kinase).^34^ More recently, a study found that EhIP6KA, an IP6K homologue from *Entamoeba histolytica*, was capable of slowly phosphorylating InsP_3_, although the products were identified as Ins(1,4,5,6)P_4_ and Ins(1,2,4,5)P_4_.^35^ On this basis, naturally occurring 1-PP-Ins(4,5)P_2_ seems unlikely. However, in both studies where the identities of the enzyme products were assigned,^34^,^35^ resistance to hydrolysis by DIPP1 was used to exclude the possibility that the products contained diphosphate groups. The present work shows that this criterion may not always be valid; in our hands, 1-PP-Ins(4,5)P_2_ was not metabolised in the presence of Mg^2+^ by any of the DIPPs, yet it does contain a diphosphate group.

Notwithstanding the evidence for PP-InsPs playing physiological roles,^4^,^5^ the present work indicates that a physiological function for 1-PP-Ins(4,5)P_2_, at least in relation to the regulation of InsP_3_ receptor-mediated Ca^2+^ release, may be unlikely. Converting the 1-phosphate of InsP_3_ into a diphosphate neither attenuates nor enhances the ability of the ligand to activate InsP_3_R. As the first example of a diphosphate analogue of a second messenger, however, the results add a new component to structure–activity relationships. Co-crystallisation studies with DIPPs using some of the non-hydrolysable substrate analogues discussed here are currently in progress.

## Experimental

### General chemistry methods

General methods were as previously reported.^36^ Alcohol **2** = 1d-2,3,6-tri-*O*-benzyl-*myo*-inositol 4,5-bis-*O*-(dibenzylphosphate) was synthesised according to the literature^19^ and crystallised from diethyl ether/light petroleum; m.p. 90–91 °C; Lit.^19^ 90–91 °C; [*α*]20D = –18.2, (*c* = 2, CHCl_3_), Lit.^19^ [*α*]25D = –15.6, (*c* = 1, CHCl_3_); Lit.^20^ [*α*]20D = –17.8, (*c* = 1.7, CHCl_3_). *N*,*N*-Diisopropylamino-bis-[2-(methylsulfonyl)ethyloxy]-phosphine (**5**) was synthesised according to the literature^24^ and recrystallized from dichloromethane/ether; m.p. 75.5–77.0 °C; ^1^H NMR (CDCl_3_, 400 MHz) *δ* 1.20 (12 H, d, ^3^*J*_HP_ 6.8 Hz, 4 × CHC*H*_3_), 3.01 (6 H, s, 2 × SCH_3_), 3.22–3.34 (4 H, m, 2 × OCH_2_C*H*_2_S), 3.59 (2 H, dh, ^3^*J*_HP_ 10.4 Hz, ^3^*J*_HH_ 6.8 Hz, 2 × C*H*CH_3_), 4.01–4.15 (4 H, m, 2 × OC*H*_2_CH_2_S); ^13^C NMR (CDCl_3_, 101 MHz) *δ* 24.62 (^3^*J*_CP_ 7.3 Hz, 4 × CH*C*H_3_), 42.85 (2 × S*C*H_3_), 43.31 (^2^*J*_CP_ 12.4 Hz, 2 × *C*HCH_3_), 56.17 (^3^*J*_CP_ 8.3 Hz, 2 × OCH_2_*C*H_2_S), 57.58 (^2^*J*_CP_ 20.0 Hz, 2 × O*C*H_2_CH_2_S); ^31^P NMR (CDCl_3_, 162 MHz, ^1^H-decoupled) *δ* 148.98; HRMS (*m*/*z*) [M + H]^+^ calcd. for C_12_H_28_O_6_NPS_2_; 378.11684; found 378.11687. 5-PP-InsP_5_, 1-PP-InsP_5_ and their PCP analogues were synthesised using similar methods to those previously described.^21^,^22^,^27^,^28^,^36^

#### d-2,3,6-tri-*O*-Benzyl-*myo*-inositol-4,5-bis(dibenzylphosphate)-1-bis[2-(methylsulfonyl)ethyl]phosphate (**3**)

To a solution of alcohol **2** (194 mg, 0.200 mmol) in dry dichloromethane (3 mL) was added 5-phenyl-1*H*-tetrazole (64 mg, 0.44 mmol) and *N*,*N*-diisopropylamino-bis-[2-(methylsulfonyl)ethyloxy]-phosphine (**5**) (130 mg, 0.344 mmol). The suspension was stirred under N_2_ at room temperature for 2 h, after which time TLC (dichloromethane : ethyl acetate 1 : 1) showed total conversion of **2** (*R*_f_ 0.56) into a more polar product (*R*_f_ 0.24). The mixture was then cooled to –78 °C, before *m*CPBA (70%, 100 mg, 0.406 mmol) was added. The mixture was allowed to warm to room temperature and then diluted with EtOAc (30 mL). The clear, colourless solution was washed with 10% aq. Na_2_SO_3_ solution (2 × 30 mL) and 1.0 mold per m^3^ HCl (30 mL), then dried over MgSO_4_ and concentrated. The residue was purified by flash chromatography on silica, eluting with methanol in ethyl acetate (0 to 15%) to give **3** as a colourless oil (225 mg, 0.178 mmole, 89%); TLC (ethyl acetate : methanol 10 : 1): *R*_f_ = 0.50; [*α*]20D = –10.3, (*c* = 1.4, CHCl_3_); ^1^H NMR (CDCl_3_, 400 MHz) *δ* 2.76 (3 H, s, SCH_3_), 2.82 (3 H, s, SCH_3_), 2.79–2.87 (1 H, m, OCH_2_C*H*CHS), 2.92–3.00 (1 H, m, OCH_2_C*H*CHS), 3.03–3.13 (2 H, m, 2 × OCH_2_CHC*H*S), 3.62 (1 H, dd, *J* 9.8, 1.9 Hz, H-3), 4.10 (1 H, dd, *J* 9.5, 9.5 Hz, H-6), 4.07–4.40 (6 H, m, H-1, H-2 and 2 × OC*H*_2_CH_2_CHS), 4.58–4.73 (6 H, m, H-5 and 2.5 AB systems of OC*H*_2_Ph), 4.82–5.03 (9 H, m, H-4 and 4 × OC*H*_2_Ph), 5.09, 5.11 (1 H, ^2^*J*_AB_ 11.9 Hz, ^3^*J*_HP_ 7.0 Hz, 0.5 ABX system of POC*H*_2_Ph), 6.95–6.97 (2 H, m, Ph), 7.09–7.40 (33 H, m, Ph); ^13^C NMR (CDCl_3_, 100 MHz) *δ* 42.34 (2 × CH_3_), 54.03 (^3^*J*_CP_ 7.7 Hz, POCH_2_*C*H_2_S), 54.20 (^3^*J*_CP_ 7.7 Hz, POCH_2_*C*H_2_S), 61.16–61.24 (overlapping signals with ^2^*J*_CP_ couplings, PO*C*H_2_CH_2_S), 69.14–69.55 (overlapping signals with ^2^*J*_CP_ couplings, PO*C*H_2_Ph), 72.64 (O*C*H_2_Ph), 74.75 (O*C*H_2_Ph), 75.08 (O*C*H_2_Ph), 75.19 (C-2), 77.78–78.12 (overlapping signals with *J*_CP_ couplings, C-1, C-3, C-4 and C-6), 78.81 (C-5), 127.32–128.36 (*C*H of Ph), 135.51 (^3^*J*_CP_ 7.4 Hz, *ipso-C* of POCH_2_*Ph*), 135.98–136.06 (overlapping signals with ^3^*J*_CP_ couplings, 3 × *ipso-C* of POCH_2_*Ph*), 137.53 (*ipso-C* of OCH_2_*Ph*), 138.13 (*ipso-C* of OCH_2_*Ph*), 138.19 (*ipso-C* of OCH_2_*Ph*); ^31^P NMR (CDCl_3_, 162 MHz) *δ* –3.37 (1 P), –1.94 (1 P), –1.59 (1 P); HRMS (*m*/*z*) [M + Na]^+^ calcd. for C_61_H_69_O_19_P_3_S_2_; 1285.2980; found 1285.3011.

#### d-1-Diphospho-*myo*-inositol 4,5-bisphosphate (**1**)

Compound **3** (63 mg, 50 μmol) was dissolved in dry CDCl_3_ (1.5 mL) and the solution was transferred to an NMR tube. A ^31^P NMR spectrum (^1^H decoupled) showed three peaks as described above. DBU (30 μL, 200 μmol), followed by BSTFA (53 μL, 200 μmol) was added and the sample was shaken to mix the liquids. A ^31^P NMR spectrum taken after 1 h now showed three peaks: *δ* –1.84 (1 P), –2.20 (1 P) and –18.10 (1 P), this last signal corresponding to the bis-silylated phosphate triester at *O*-1. Methanol (100 μL) was added and the tube was shaken again. After 10 min, TFA (15 μL, 200 μmol) was added and the solution was concentrated by evaporation under reduced pressure, then thoroughly dried under vacuum. A ^31^P NMR spectrum (CDCl_3_) of the residue showed that the silyl groups were completely cleaved, with three peaks at *δ* –0.18 (1 P, P-1), –2.23 (1 P) and –2.45 (1 P). To this residue was added 5-phenyl-1*H*-tetrazole (20 mg, 137 μmol). Then, under argon, dry dichloromethane (2 mL) followed by bis(benzyloxy)diisopropylaminophosphine (30 μL, 89 μmol) were added. The mixture was stirred under argon for 45 min, after which time a ^31^P NMR spectrum of a sample (CDCl_3_ added) showed major peaks at *δ* 127.24 (^2^*J*_PP_ 4.2 Hz, P-1_β_), 7.59 (H-phosphonate by-product from hydrolysis of excess P(iii) reagent), –2.22 and –2.41 (P-4 and P-5) and –10.26 (^2^*J*_PP_ 4.2 Hz, P-1_α_). The solution was cooled to –78 °C and *m*CPBA (70%, 25 mg, 100 μmol) was added. After 5 min, the solution was allowed to warm to room temperature, then concentrated under reduced pressure (no heat). A ^31^P NMR spectrum of the residue now showed peaks at *δ* 7.62 (H-phosphonate by-product), –2.28 and –2.52 (P-4 and P-5) –11.97 (d, ^2^*J*_PP_ 14.7 Hz, P-1_β_) and –13.69 (d, ^2^*J*_PP_ 14.7 Hz, P-1_α_). This residue was purified by flash chromatography on silica (methanol in ethyl acetate, 0 to 20%) giving **4** as a colourless oil (59 mg, 45 μmol, 90%); TLC (ethyl acetate : methanol 10 : 1): *R*_f_ = 0.30; ^31^P NMR (CDCl_3_, 162 MHz, ^1^H-decoupled) *δ* –1.75 (1 P, s), –2.35 (1 P, s), –11.08 (1 P, broad s, P-1_β_), –12.32 (1 P, broad s, P-1_α_); HRMS (*m*/*z*) [M + Na]^+^ calcd. for C_69_H_70_O_18_P_4_; 1333.3405; found 1333.3377. In earlier trials, this material had been found to be unstable after flash chromatography; a portion of it was therefore deprotected immediately as follows.

Compound **4** (37 mg, 28 μmol) was dissolved in methanol (4 mL) and deionised water (1 mL). Powdered NaHCO_3_ (14 mg, 168 μmol) was added followed by Pd(OH)_2_/C (30 mg). The suspension was stirred vigorously under H_2_ (balloon) for 24 h, after which time more water (4 mL) was added. A fresh balloon of H_2_ was attached and stirring was continued for a further 72 h. The catalyst was then removed by filtration through a PTFE filter, giving a colourless solution, which was concentrated under reduced pressure to give a solid white residue. Analysis of this residue by ^31^P and ^1^H NMR in D_2_O showed that deprotection was complete. The residue was purified by anion-exchange chromatography on Q-Sepharose Fast Flow resin, eluting with a gradient of 0 to 1.5 M triethylammonium bicarbonate (TEAB). The target compound **1** eluted at 70 to 77% 1.5 M TEAB. Fractions containing the target were identified using the Briggs phosphate assay, combined and evaporated under reduced pressure. De-ionised water was repeatedly added and evaporated until the triethylammonium salt of **1** remained as a colourless glassy solid (14 mg, 16 μmol, 57%). This material was accurately quantified using total phosphate assay^37^ before biological evaluation. For ^31^P and ^1^H NMR analysis of **1**, a small amount of EDTA (sodium salt, approx. 0.1 mg) was added to a sample of **1** (2.0 μmole in 0.4 mL D_2_O) to give sharper signals. This NMR sample containing EDTA was kept as the solution in D_2_O for >1 year at 4 °C with no sign of deterioration. ^1^H NMR (D_2_O, 500 MHz, EDTA added) *δ* 3.77 (1 H, dd, *J* 9.8, 2.9 Hz, H-3), 3.95 (1 H, t, *J* 9.6 Hz, H-6), 4.08 (1 H, apparent q, *J* 9.1 Hz, H-5), 4.15 (1 H, ddd, *J* 9.9, 8.3, 2.8 Hz, H-1), 4.32 (1 H, apparent q, *J* 9.4 Hz, H-4), 4.35 (1 H, apparent t, *J* 2.8 Hz, H-2); ^13^C NMR (D_2_O, 101 MHz) *δ* 70.15 and 70.31 (C-2 and C-3), 70.88 (C-6), 76.28 (^2^*J*_CP_ 5.6 Hz, C-1), 76.82 (with *J*_CP_ couplings, C-4) and 78.11 (with *J*_CP_ couplings, C-5); ^31^P NMR (D_2_O, 202 MHz, EDTA added, ^1^H-decoupled) *δ* 1.11 (1 P), 0.45 (1 P), –10.48 (1 P, d, *J* 20.9 Hz, P-1_β_), –11.96 (d, *J* 20.9 Hz, P-1_α_); ^31^P NMR (D_2_O, 162 MHz, EDTA added, ^1^H-coupled) *δ* 1.13 (1 P, d, ^3^*J*_HP_ 8.8 Hz), 0.47 (1 P, d, ^3^*J*_HP_ 8.9 Hz), –10.46 (1 P, d, ^2^*J*_PP_ 20.5 Hz, P-1_β_), –11.94 (1 P, dd, ^2^*J*_PP_ 20.5, ^3^*J*_HP_ 8.3 Hz, P-1_α_); HRMS (*m*/*z*) M^–^ calcd. for C_6_H_16_O_18_P_4_; 498.9209; found 498.9214.

#### Molecular docking of 1-PP-Ins(4,5)P_2_ (**1**) into type 1 InsP_3_ receptor

Molecular docking experiments were carried out using the X-ray crystal structure of the *N*-terminal IBC of type 1 InsP_3_ receptor in complex with Ins(1,4,5)P_3_ (1N4K).^18^ Docking methods were optimised by docking flexible models of Ins(1,4,5)P_3_ into the 1N4K structure using GOLD^38^ (version 5.6, CCDC). In the most successful protocol, the binding site was defined as a sphere of 6 Å radius centred on the centroid of bound Ins(1,4,5)P_3_ and two water molecules (waters 1139 and 1198) were included in the docking protocol. These water molecules were toggled on and off and allowed to spin in the docking runs.^39^ The ligand was docked 100 times using the GoldScore scoring function, and genetic algorithm settings for very flexible ligands were used. This method accurately reproduced the observed pose of bound Ins(1,4,5)P_3_ in 1N4K; the ten highest scoring poses all closely resembled the conformation of bound Ins(1,4,5)P_3_ (mean RMSD 0.58 Å). When 1-PP-Ins(4,5)P_2_ (**1**) was docked using the same protocol, the highest-scoring poses were very similar to the bound conformation of Ins(1,4,5)P_3_ but often showed additional interactions of the 1-beta-phosphate group with residues in the binding site. More details are given in the ESI.†

#### Assays of InsP_3_ receptor activity

Ca^2+^ release from the intracellular stores of permeabilised DT40 cells expressing rat type 1 InsP_3_ receptors was measured in cytosol-like medium (CLM) using a low-affinity fluorescent Ca^2+^ indicator trapped within the endoplasmic reticulum as previously reported.^40^ Equilibrium competition binding of [^3^H]-InsP_3_ (1.5 nM, 19.3 Ci mmol^–1^) to membranes prepared from insect Sf9 cells expressing rat type 1 InsP_3_ receptors was determined in CLM at 4 °C. Bound and free ligand were separated by centrifugation and non-specific binding was determined by addition of 10 μM InsP_3_.

#### DIPP purification

cDNAs for all DIPPs were kind gifts from the Structural Genomics Consortium, Stockholm. cDNAs were modified as necessary in order to represent the full-length constructs, cloned into pET28a (+) and expressed as *N*-terminally His-tagged proteins. All proteins were expressed in BL21 (DE3) T1R pRARE2 at 18 °C overnight and purified by the Protein Science Facility (PSF) at the Karolinska Institute, Stockholm. Briefly, the proteins were first purified over a HisTrap HP column (GE Healthcare), followed by thrombin cleavage of the *N*-terminal His-tag. After removal of the His-tag through a second run over a HisTrap HP column, the proteins were further purified by gel filtration using a HiLoad 16/60 Superdex 75 column (GE Healthcare).

#### Enzyme activity assay (DIPPs)

Activity of DIPPs with a panel of potential substrates (1-PP-InsP_5_, 5-PP-InsP_5_, 1-PP-Ins(4,5)P_2_ (**1**), Ap_3_A, and Ap_5_A (Sigma Aldrich)) and control compounds (1-PCP-InsP_5_ and 5-PCP-InsP_5_) was assessed in technical triplicates in reaction buffer (100 mM Tris acetate, pH 7.5, 40 mM NaCl, 1 mM DTT) containing either 1 mM Mg acetate or MnCl_2_. Following an incubation time of 20 min at room temperature with shaking, the formed inorganic phosphate was detected through addition of malachite green reagent.^41^ After an additional 15 min incubation with shaking, absorbance at 630 nm was read using a Hidex Sense plate reader.

#### Differential scanning fluorimetry (DSF)

DSF^42^ was performed with 5 μM purified protein in 25 mM HEPES pH 7.5, 100 mM NaCl, 0.5 mM TCEP and 5× Sypro Orange added per well of a 96-well PCR plate. Substrates and substrate analogues were dissolved in water and diluted 1 : 10 in the assay mixture. The heat denaturation curves with a temperature increase of 1 °C min^–1^ from 25 °C to 95 °C were recorded on a CFX96 real-time PCR machine (Bio-Rad) by measuring the fluorescence of Sypro Orange with excitation and emission wavelengths of 470 and 570 nm, respectively. The Boltzmann equation was used to analyse the denaturation curves in GraphPad Prism. The determined melting temperature (*T*_m_) is the inflection point of the sigmoidal denaturation curve.

## Conflicts of interest

There are no conflicts to declare.

## Supplementary Material

Supplementary information
