# Supplementary material for: A synthetic diphosphoinositol phosphate analogue of inositol trisphosphate
Source: Medchemcomm. 2018 Jun 4;9(7):1105–13. doi: 10.1039/c8md00149a (PMC6071853; doi:10.1039/c8md00149a)
Supplement: Supplementary file 1 [file MD-009-C8MD00149A-s001.pdf]

Electronic Supplementary Material (ESI) for MedChemComm.
This journal is © The Royal Society of Chemistry 2018

Supplementary Information

A synthetic diphosphoinositol phosphate analogue of inositol trisphosphate

Andrew M. Riley,[a] Judith E. Unterlass,[b] Vera Konieczny,[c] Colin W. Taylor,[c] Thomas Helleday[b] and Barry V. L. Potter[a]

[a]*Medicinal Chemistry & Drug Discovery, Department of Pharmacology, University of Oxford, Mansfield Road, Oxford OX1 3QT.*

[b]*Science for Life Laboratory, Department of Oncology-Pathology, Karolinska Institutet, SE-171 21 Solna, Sweden.*

[c]Department *of Pharmacology, University of Cambridge, Tennis Court Road, Cambridge CB2 1PD, UK.*

Table of Contents

Molecular Docking Experiments S2-S5

Stabilisation of DIPPs by 1-PP-InsP$_5$ and 1-PCP-InsP$_5$ S6

Stabilisation of DIPPs by 5-PP-InsP$_5$ and 5-PCP-InsP$_5$ S7

NMR Spectra S8-S15

Additional References S16

Molecular Docking

Molecular docking experiments were carried out using the GOLD Suite[1] (version 5.6, CCDC) and the X-ray crystal structure of Type 1 $InsP_3$ receptor in complex with Ins(1,4,5)P_3 (1N4K)[2]. Docking methods were optimised by docking flexible models of Ins(1,4,5)P_3 into the 1N4K structure. The ligands were built in Chem3D (PerkinElmer) with fully charged phosphate groups and subjected to an initial minimisation step using the MMFF94 force field. The geometry of models was then checked using the Mogul Geometry Check module of Mercury (Version 3.10, CCDC) after a further minimisation step using the CSD Conformer Generator *via* the Mercury interface.

The binding site was defined in GOLD as a sphere of 6 Å radius centred on the centroid of bound Ins(1,4,5)P_3. Two water molecules (water 1139 and 1198) were included in the docking protocol. These water molecules were toggled on and off and allowed to spin in the docking runs.[3] Our reason for including these water molecules in the docking protocol was that, in the 1N4K structure, they bridge interactions between the 1-phosphate group of bound Ins(1,4,5)P_3 and residues in the binding site. Thus, modifications to the 1-phosphate group, as in 1-PP-Ins(4,5)P_2, might be expected to perturb these interactions, e.g. by displacing one or both water molecules. GOLD attempts to take this into account by calculating the free-energy change associated with transferring each water molecule from bulk solvent into its binding site.

Each ligand was docked 100 times using the GoldScore scoring function. Genetic algorithm settings for very flexible ligands were used. Using Ins(1,4,5)P_3, this protocol accurately reproduced the observed pose of bound Ins(1,4,5)P_3 in 1N4K; the ten highest scoring poses all closely resembled the conformation of bound Ins(1,4,5)P_3 (mean RMSD 0.58 Å). In a control experiment, the inactive L-isomer of Ins(1,4,5)P_3 [=Ins(3,5,6)P_3] was docked into the 1N4K structure using the same protocol. In this case, the highest scored poses of L-$InsP_3$ did not resemble the bound conformation of Ins(1,4,5)P_3, and were much lower scored by the GoldScore function. When 1-PP-Ins(4,5)P_2 (**1**) was docked using the same protocol, the highest-scoring poses were very similar to the bound conformation of Ins(1,4,5)P_3 but often showed additional interactions of the 1-beta-phosphate group with residues in the binding site.

Using the GOLD scoring function, the highest scored poses of 1-PP-Ins(4,5)P_2 (**1**) had higher "fitness" scores than the best poses of Ins(1,4,5)P_3, which might be taken to predict higher binding affinity of 1-PP-Ins(4,5)P_2 (**1**) for InsP_3R. However, while fitness scores in GOLD may have some relation to binding affinities, the GOLDScore function is optimised for accurate pose prediction, rather than as a predictor of relative binding affinities between ligands. Indeed, we have found that GOLDScore generally gives higher fitness scores for more highly phosphorylated ligands, e.g. Ins(1,3,4,5,6)P_5, which, nevertheless, are known to have lower affinities for InsP_3 receptors than InsP_3. This may be because Goldscore heavily rewards hydrogen bonds involving charged groups. We therefore used the Rescore option in GOLD to re-score the docked poses with ChemScore, using the Receptor Depth Scaling option available with this function. Using receptor depth scaling. the score attributed to hydrogen bonds is scaled depending on the depth in the pocket. Hydrogen bonds deep in the pocket are rewarded with an increased score, while the scores of those closer to the solvent-exposed surface are decreased. We found that, while both GOLDScore and ChemScore (with receptor depth scaling) were substantially in agreement on the predicted binding poses for Ins(1,4,5)P_3 and 1-PP-Ins(4,5)P_2, GOLDScore scored the top poses for 1-PP-Ins(4,5)P_2 more highly than those for Ins(1,4,5)P_3, while the reverse was true with ChemScore. The two scoring functions did not agree on predicted binding poses for L-Ins(1,4,5)P_3 (which in reality binds only very weakly) although both functions scored L-Ins(1,4,5)P_3 much lower than either D-Ins(1,4,5)P_3 or 1-PP-Ins(4,5)P_2.

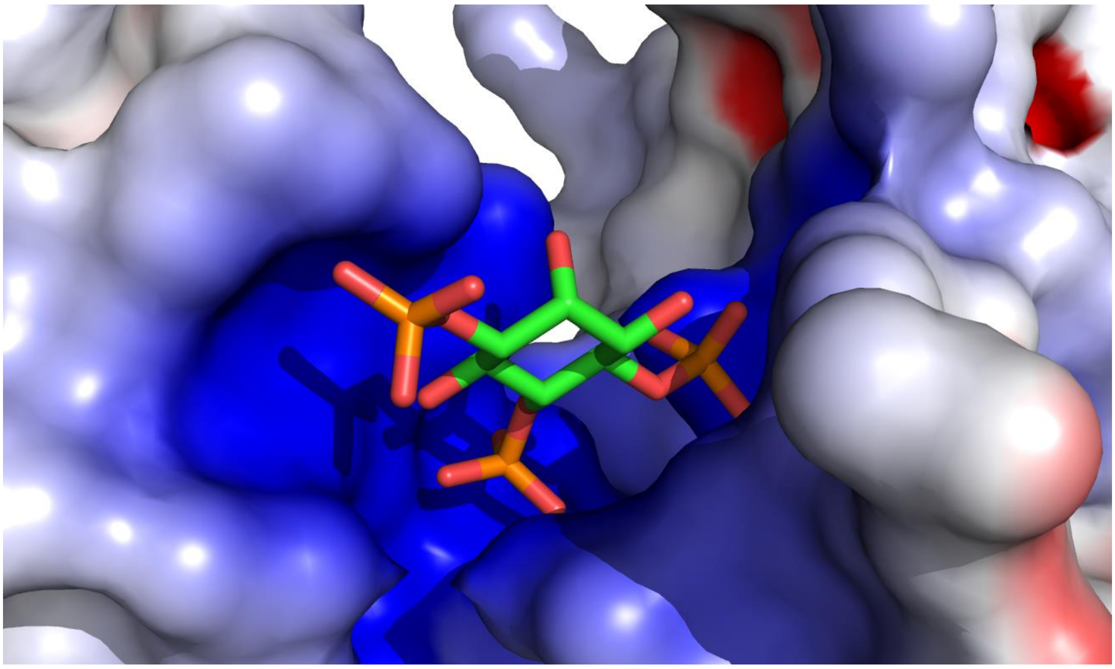

Figure 1S. $InsP_3$ in the binding site of Type 1 $InsP_3$ receptor based on the X-ray structure of $InsP_3$ in complex with the IBC (1N4K)[2]. The protein is shown as a solvent-accessible surface, coloured by electrostatic potential using APBS Tools within Pymol.

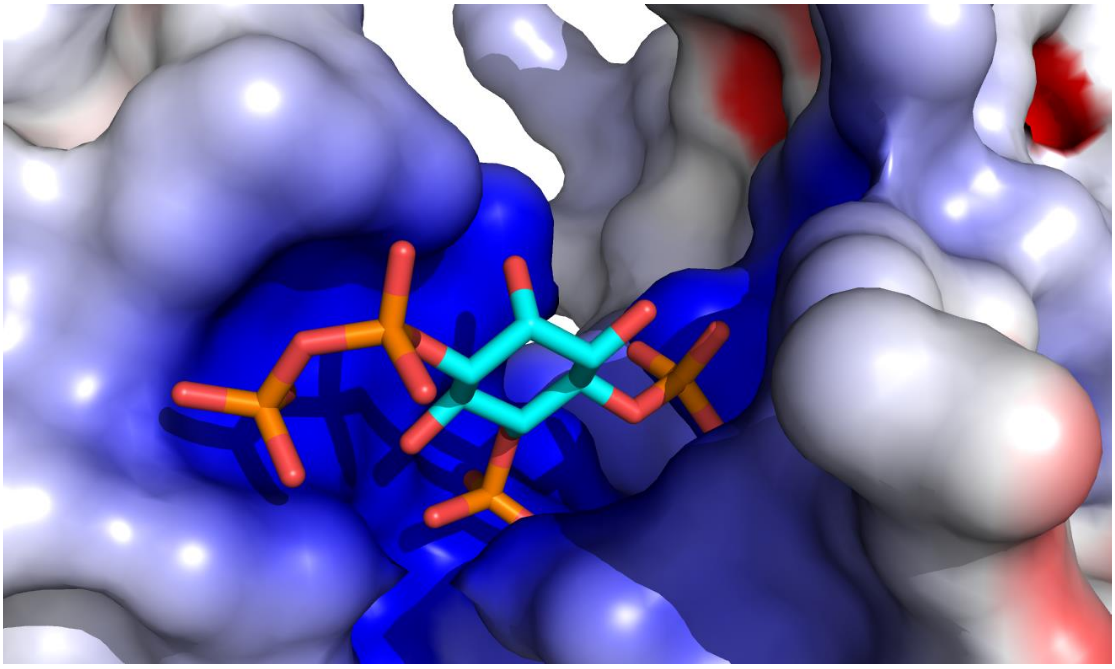

Figure 2S. Highest scoring pose of 1-PP-Ins(4,5)P_2 (**1**) docked into the $InsP_3$-binding site using the 1N4K structure and GOLD docking protocol as described.

D-Ins(1,4,5)P_3

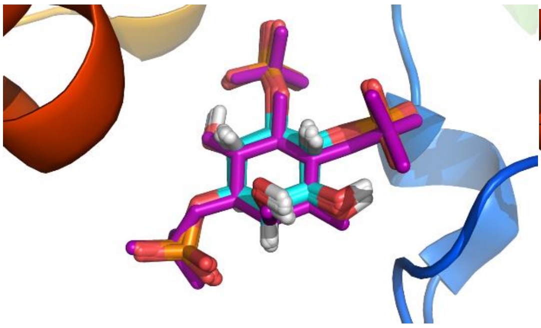

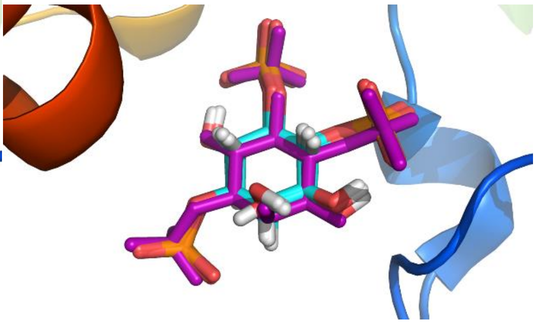

GOLD
Max. fitness score 115.5

Rescored with ChemScore
Max. fitness score 23.4

L-Ins(1,4,5)P_3

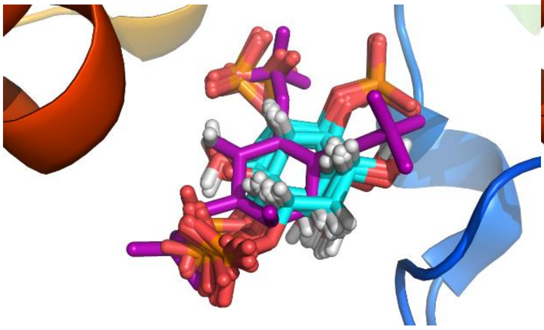

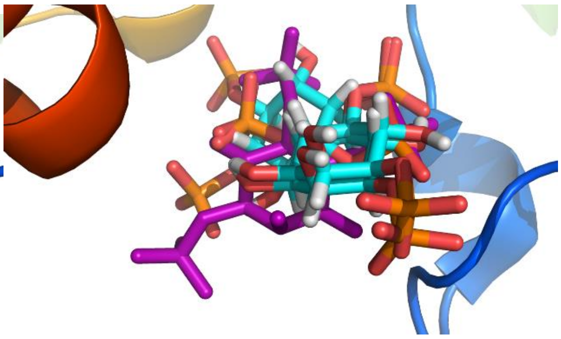

GOLD
Max. fitness score 96.5

Rescored with ChemScore
Max. fitness score 10.1

1-PP-Ins(4,5)P_2

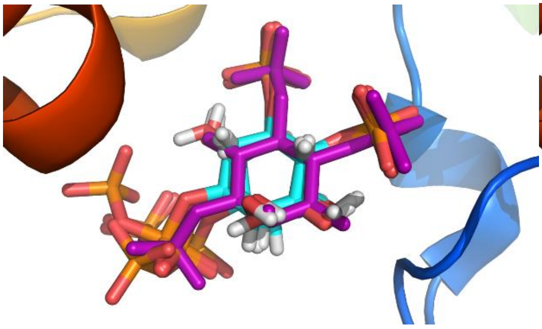

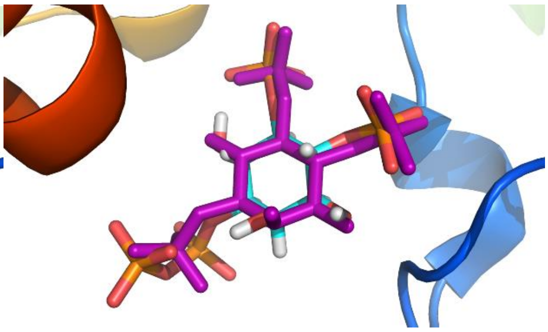

GOLD
Max. fitness score 122.2

Rescored with ChemScore
Max. fitness score 16.2

Figure 3S. Highest scored poses of InsP_3, L-InsP_3 and 1-PP-Ins(4,5)P_3 (**1**) in the InsP_3-binding core (IBC) of type 1 InsP_3 receptors, predicted by molecular docking. The crystallographic pose of bound InsP_3 taken from 1N4K[2] is shown as a purple stick model.

Stabilisation of DIPPs by 1-PP-InsP$_5$ and 1-PCP-InsP$_5$

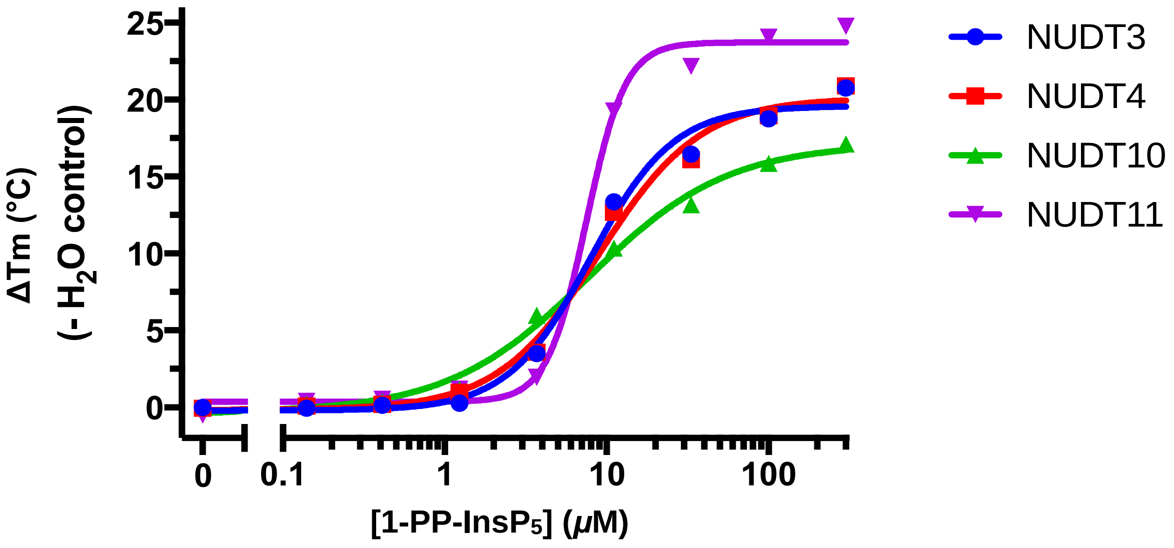

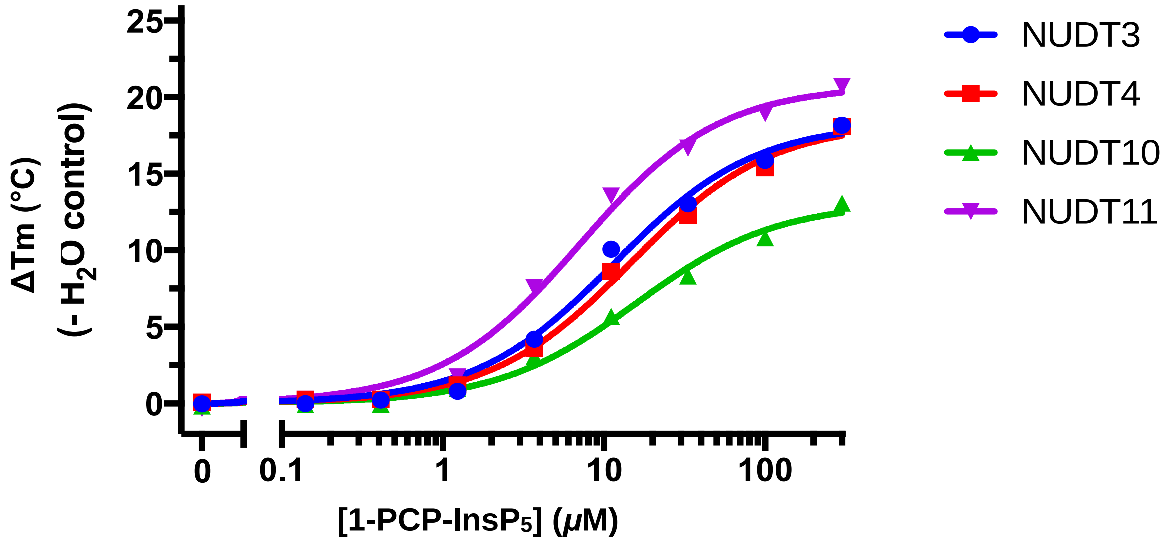

Figure 4S. Dose-response curves for stabilisation of DIPPs by 1-PP-InsP$_5$ and 1-PCP-InsP$_5$, measured by differential scanning fluorimetry (DSF).

Table 1S. K_d values (μM) calculated from dose-response curves in Figure 4S.

	K_d (μM)			
	**NUDT3**	**NUDT4**	**NUDT10**	**NUDT11**
**1-PP-InsP$_5$**	10.9 ± 1.2	11.3 ± 1.1	8.2 ± 1.1	9.6 ± 1.3
**1-PCP-InsP$_5$**	11.6 ± 1.1	14.6 ± 1.1	15.9 ± 1.1	7.1 ± 1.1

Stabilisation of DIPPs by 5-PP-InsP$_5$ and 5-PCP-InsP$_5$

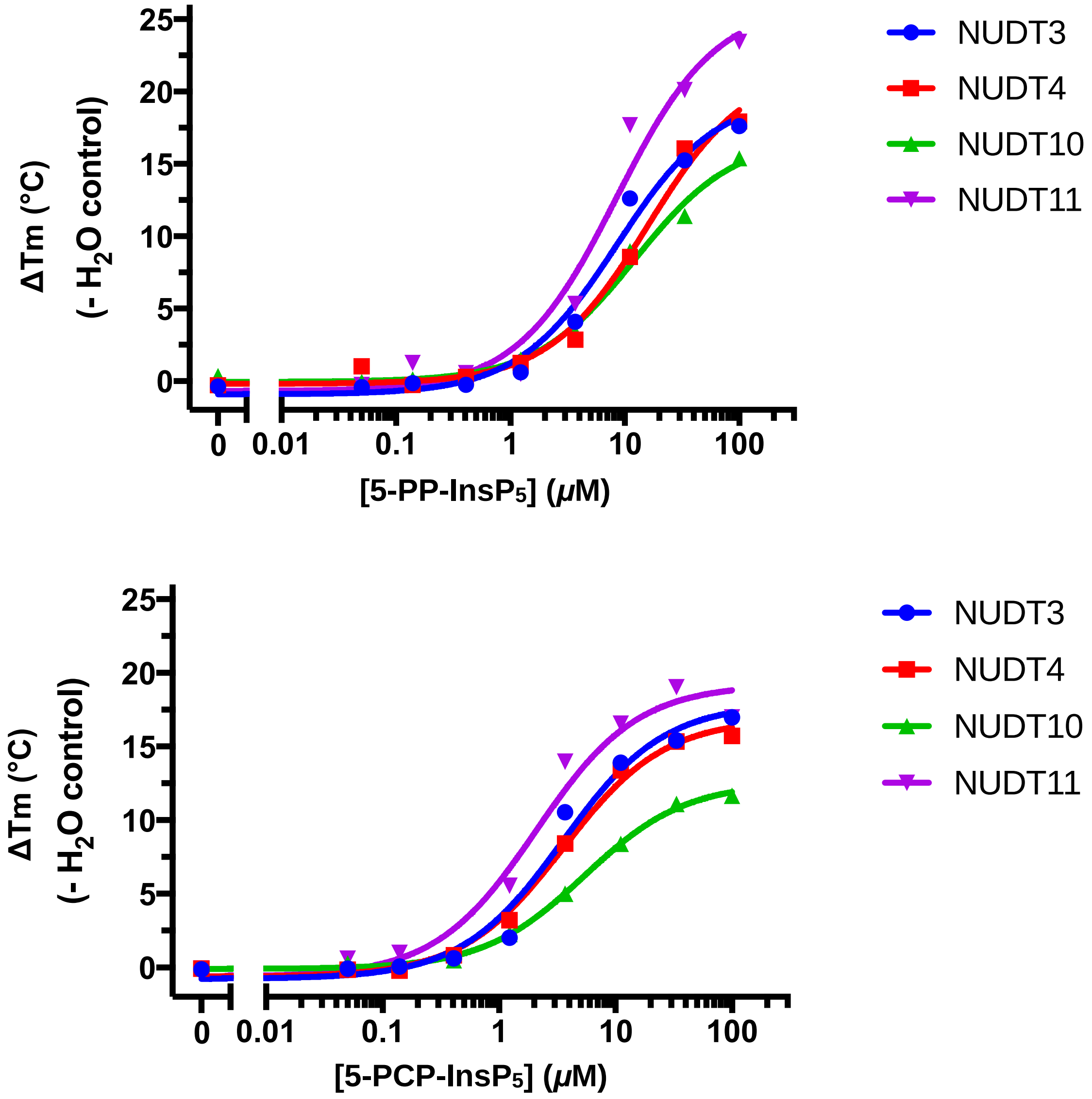

Figure 5S. Dose-response curves for stabilisation of DIPPs by 5-PP-InsP$_5$ and 5-PCP-InsP$_5$, measured by differential scanning fluorimetry (DSF).

Table 2S. K_d values (μM) calculated from dose-response curves in Figure 5S.

	K_d (μM)			
	**NUDT3**	**NUDT4**	**NUDT10**	**NUDT11**
**5-PP-InsP$_5$**	8.6 ± 1.2	16.0 ± 1.4	12.2 ± 1.2	8.5 ± 1.3
**5-PCP-InsP$_5$**	3.5 ± 1.2	3.6 ± 1.1	5.4 ± 1.1	2.1 ± 1.3

NMR Spectra

Compound **5**; ^{1}H NMR (400 MHz, $CDCl_3$)

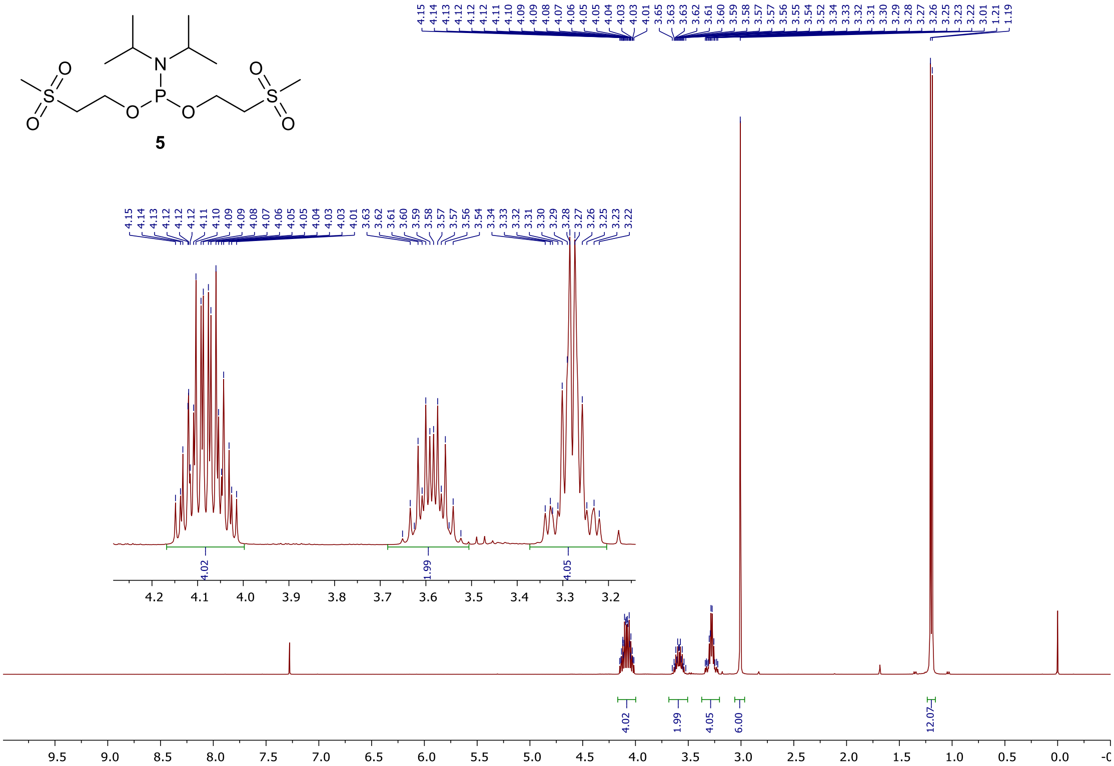

Compound **5**; ^{13}C NMR (101 MHz, $CDCl_3$)

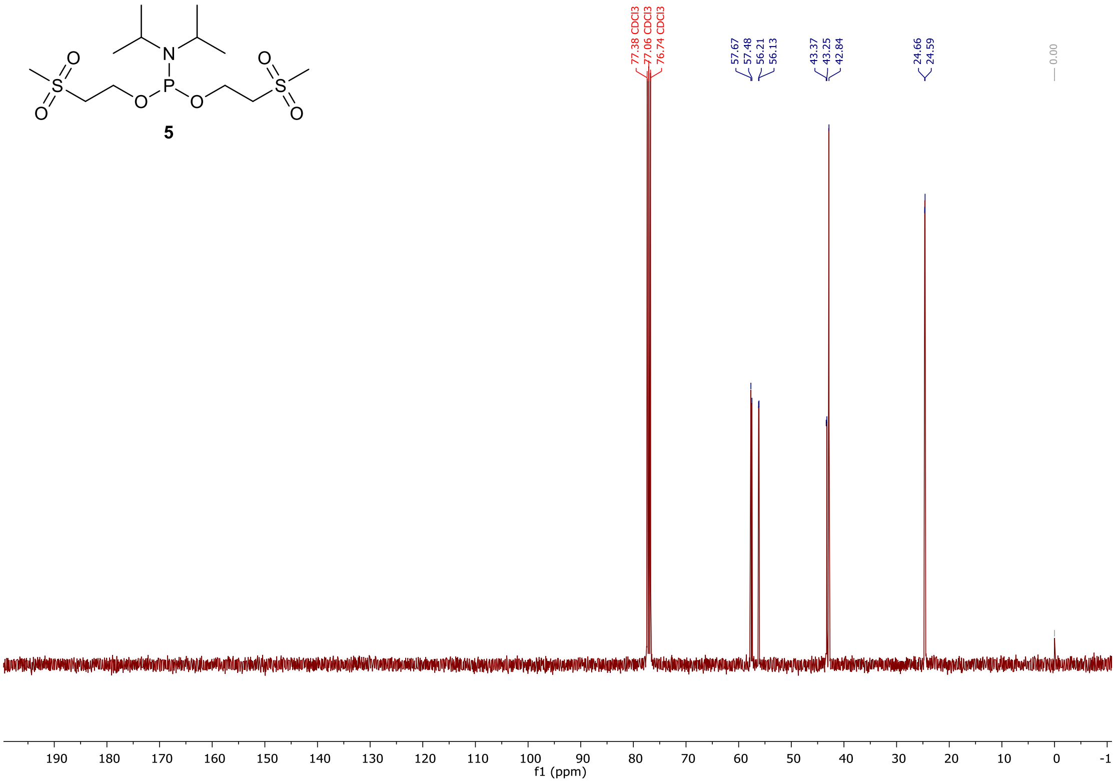

Compound **5**; ^{31}P NMR (162 MHz, $CDCl_3$, ^{1}H-decoupled)

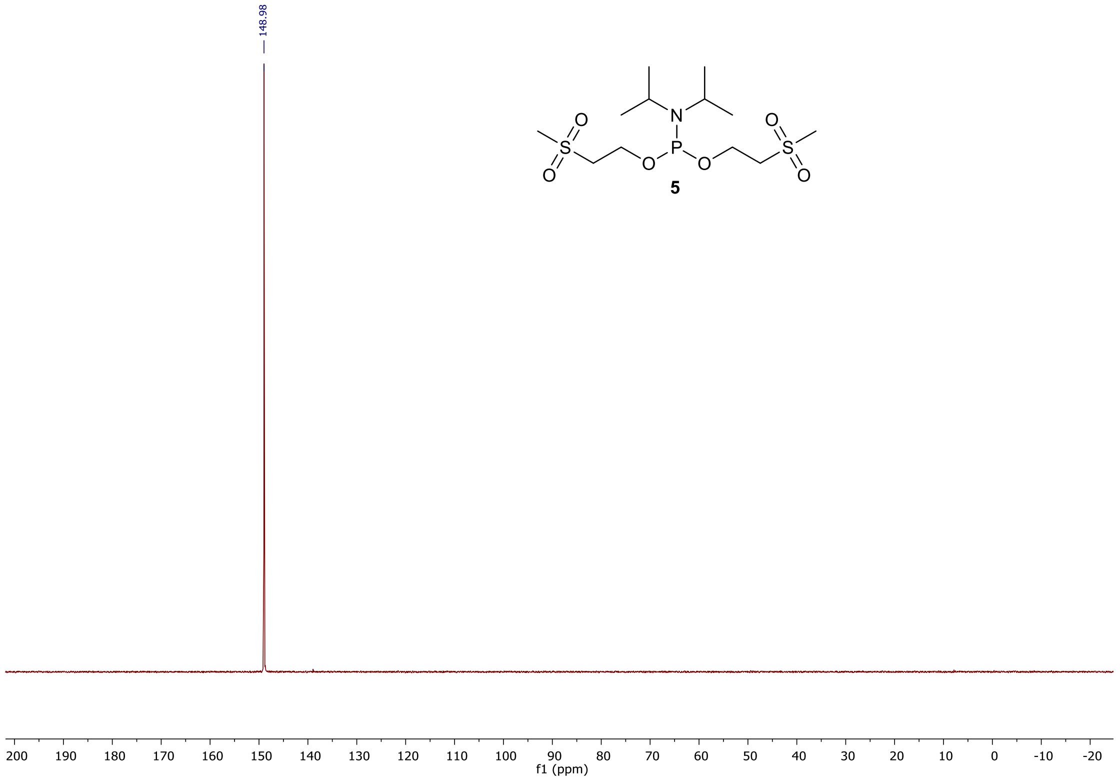

Compound **3**; ^{1}H NMR (400 MHz, $CDCl_3$)

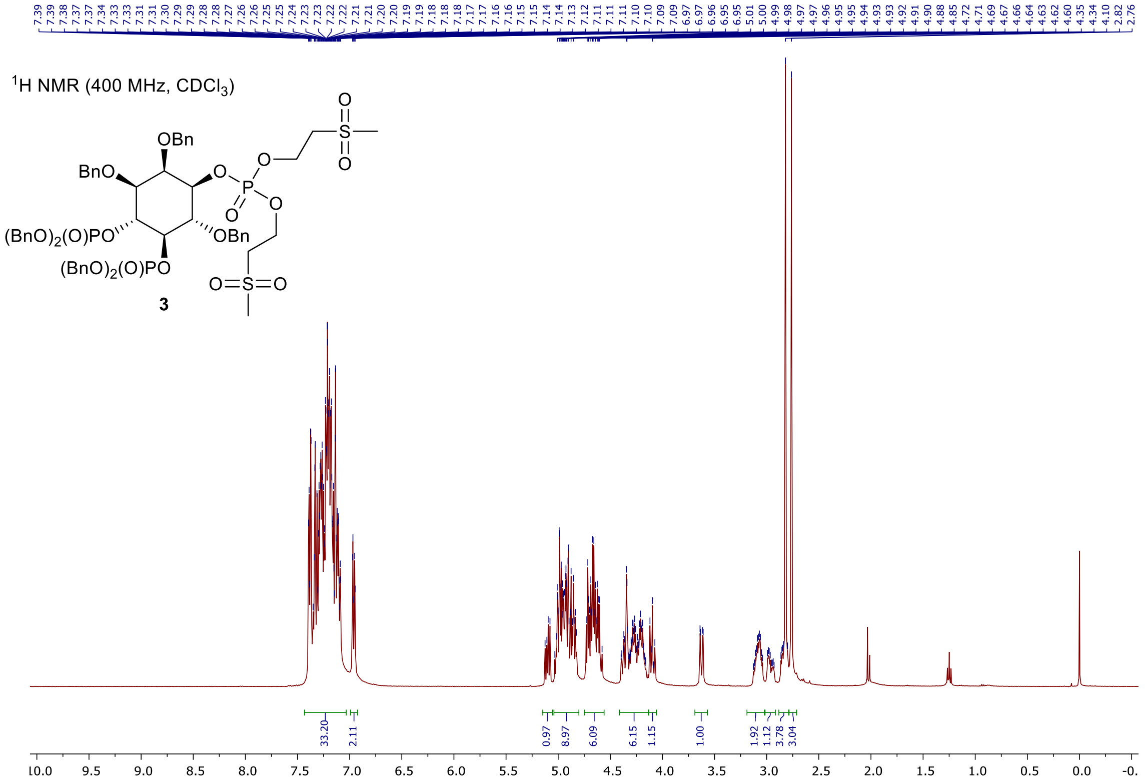

Compound **3**; ^{13}C NMR (101 MHz, $CDCl_3$)

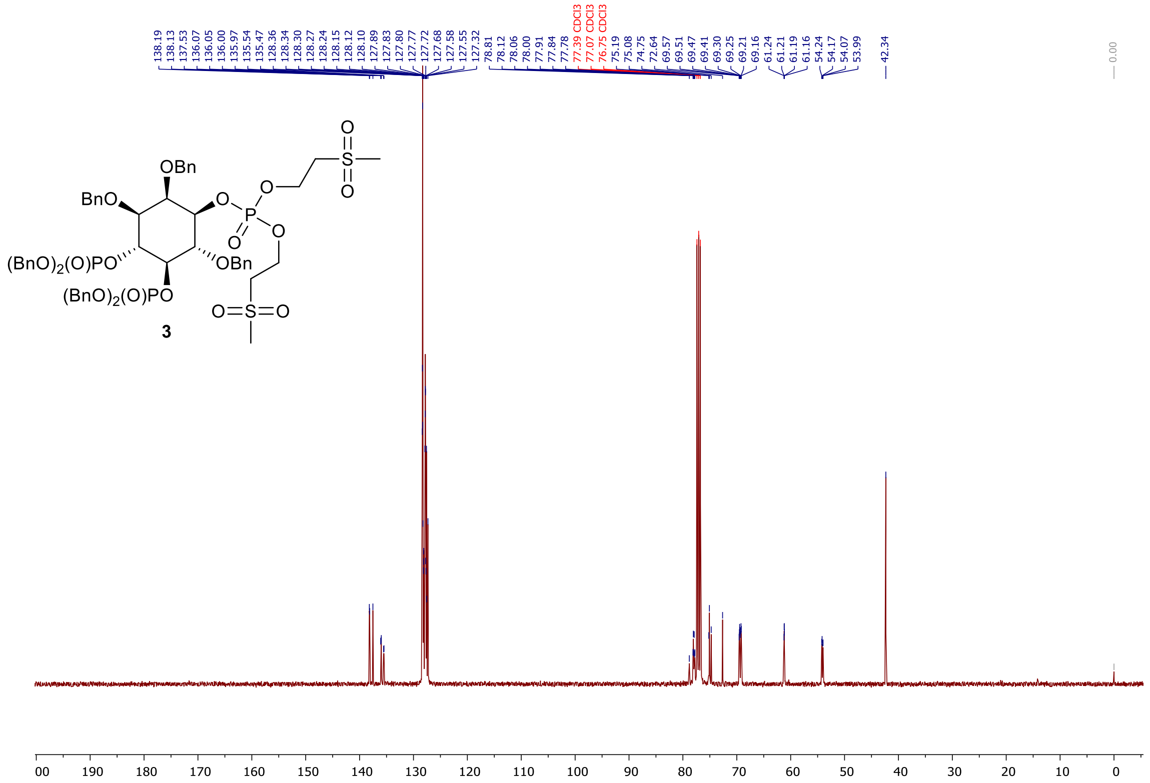

Compound **3**; ^{31}P NMR (162 MHz, $CDCl_3$, ^{1}H-decoupled)

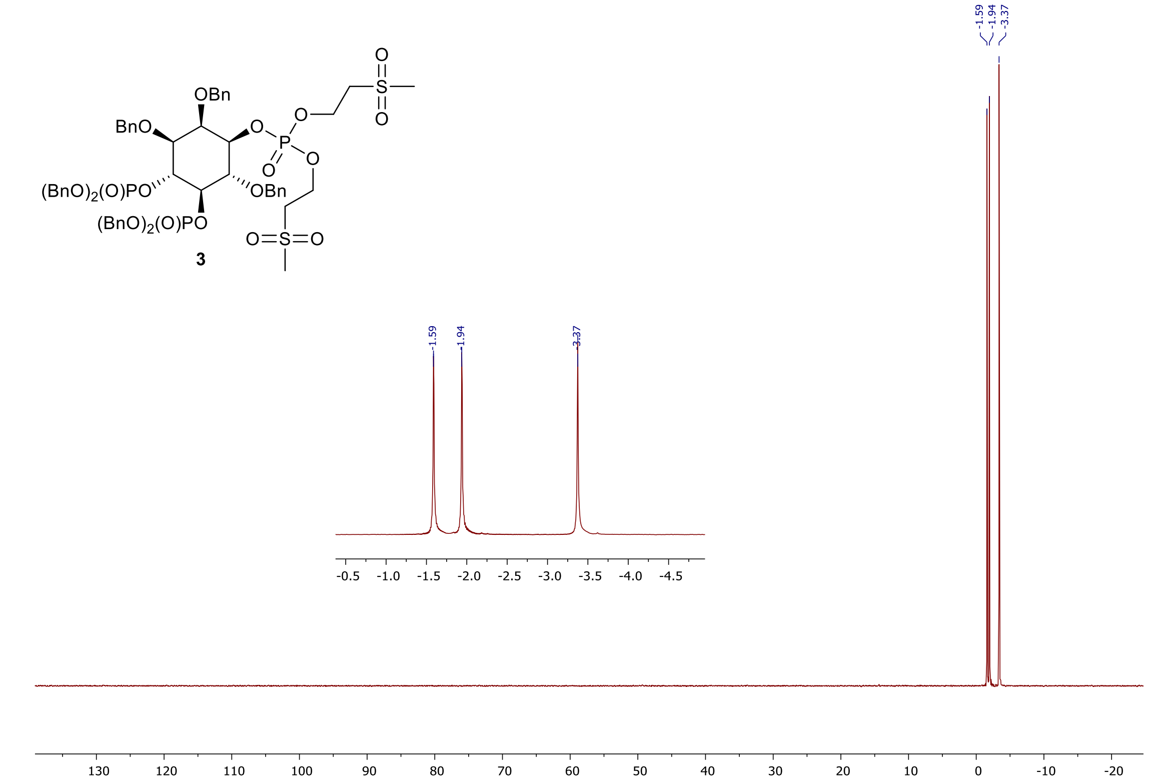

Multi-step conversion of **3** into **4** monitored by ^{31}P NMR (162 Mz, $CDCl_3$, ^1H-decoupled)

Compound 3

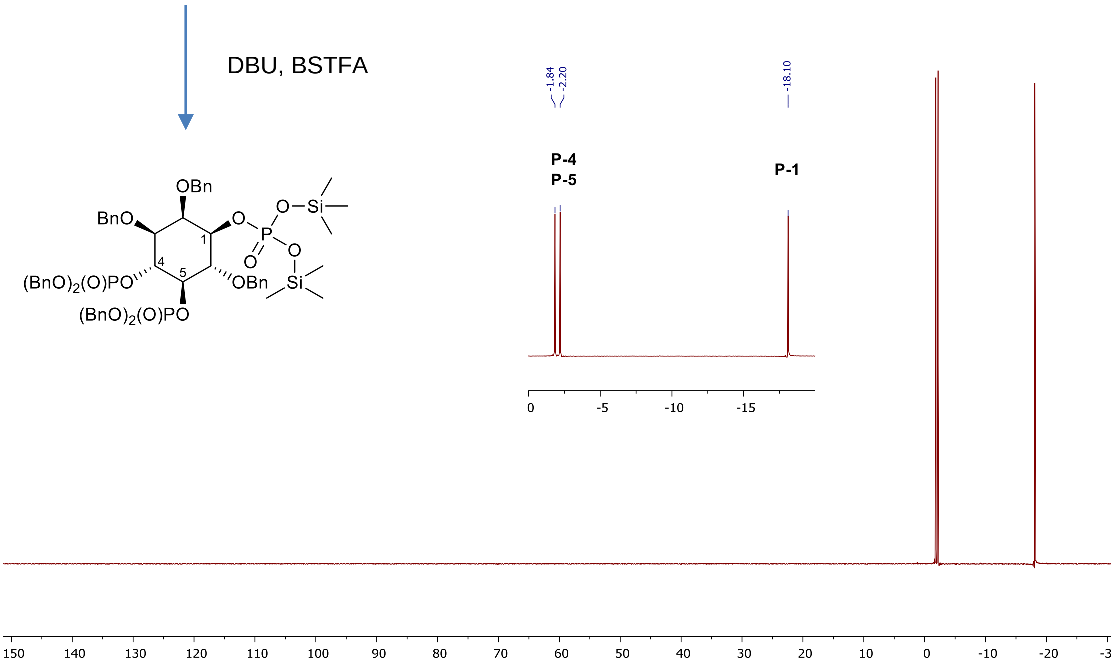

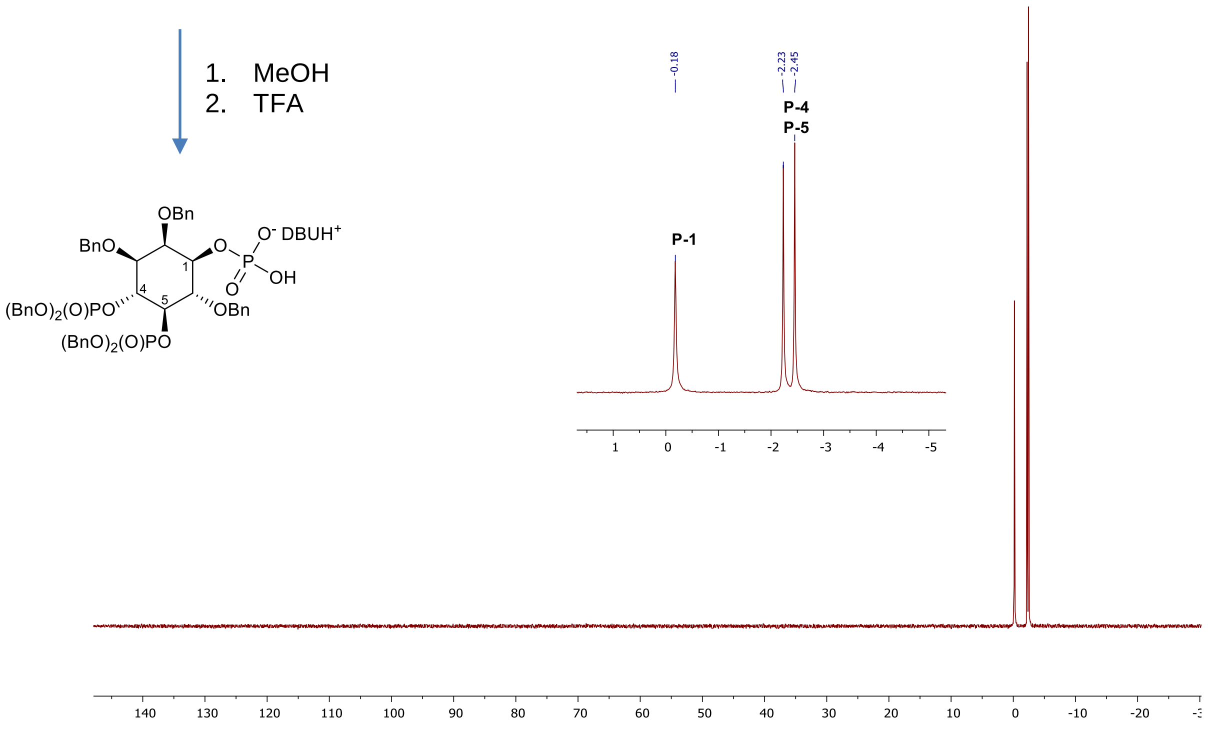

Note: TFA should be added *after* methanol (see Experimental). Otherwise it greatly slows the removal of the second TMS group, seen as a persisting signal at –8.23 ppm, even after several hours at room temp.

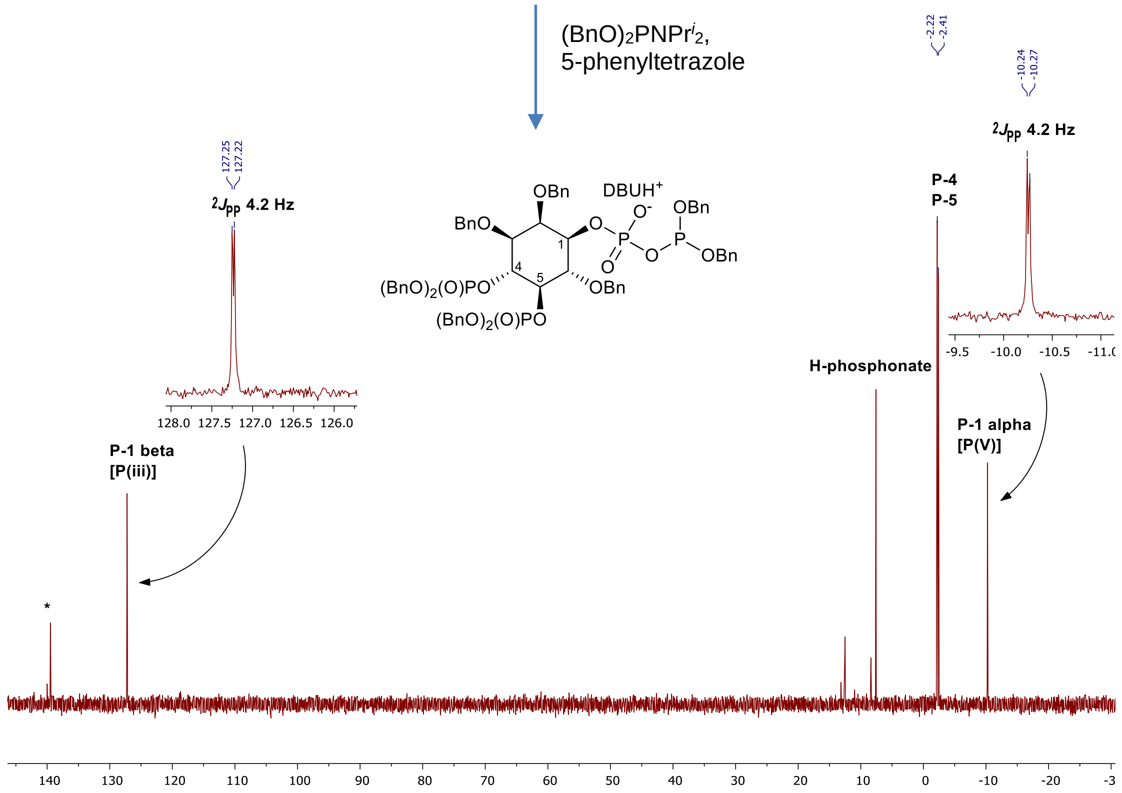

*(BnO)$_3$P (δ_P 139.48) present as an impurity in commercially available (BnO)$_2$PNPr$^i_2$

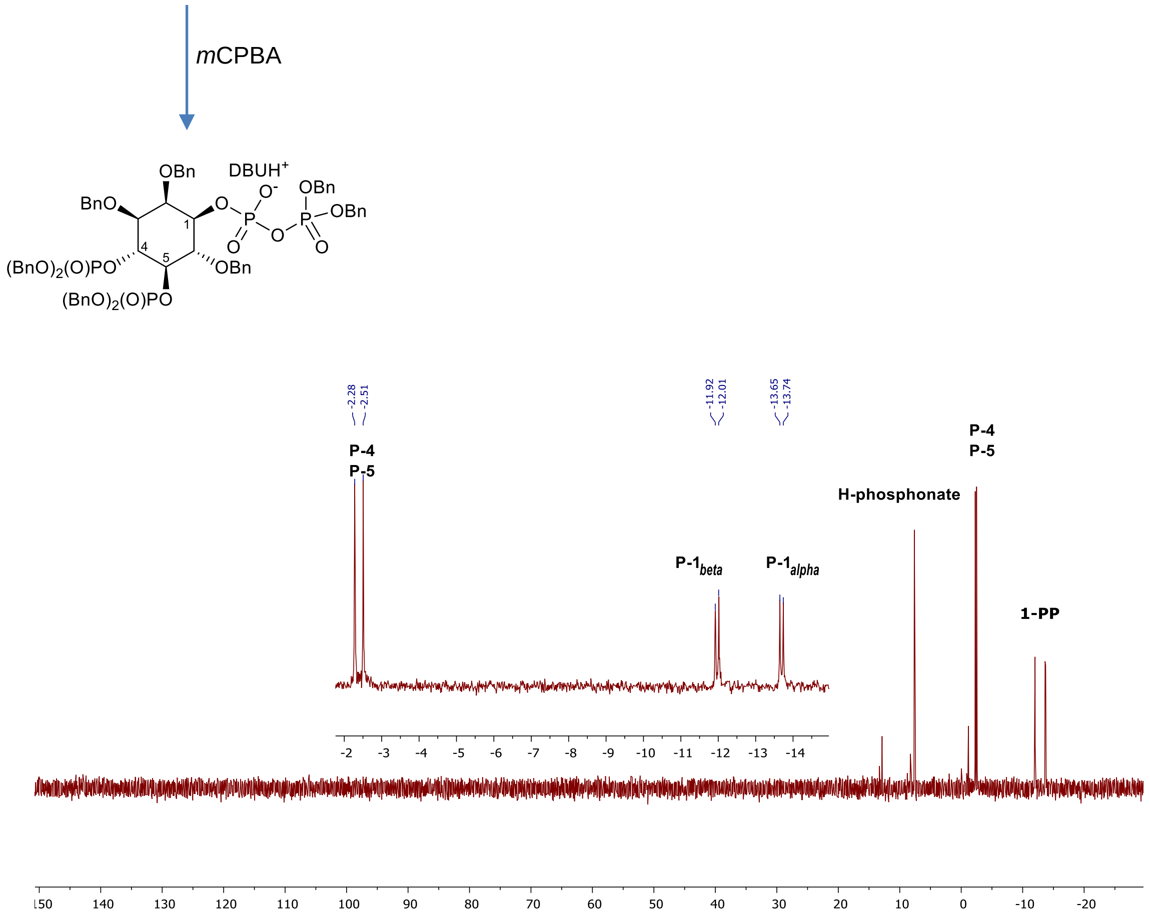

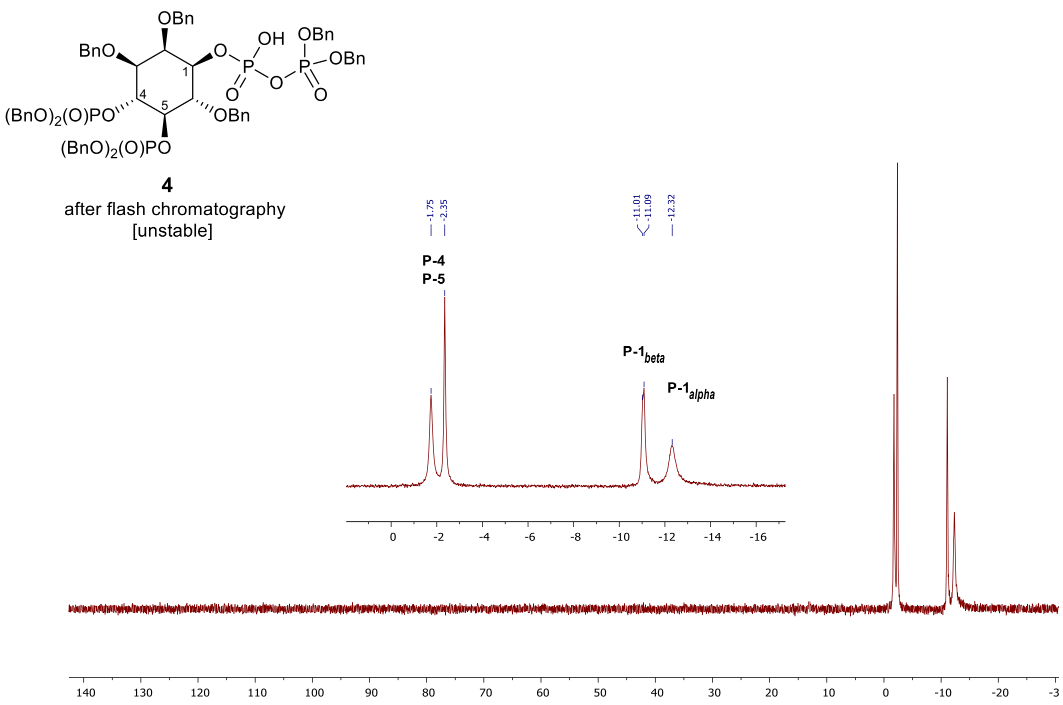

1-PP-Ins(4,5)P$_2$ (1) ^{1}H NMR (500 MHz, D$_2$O)

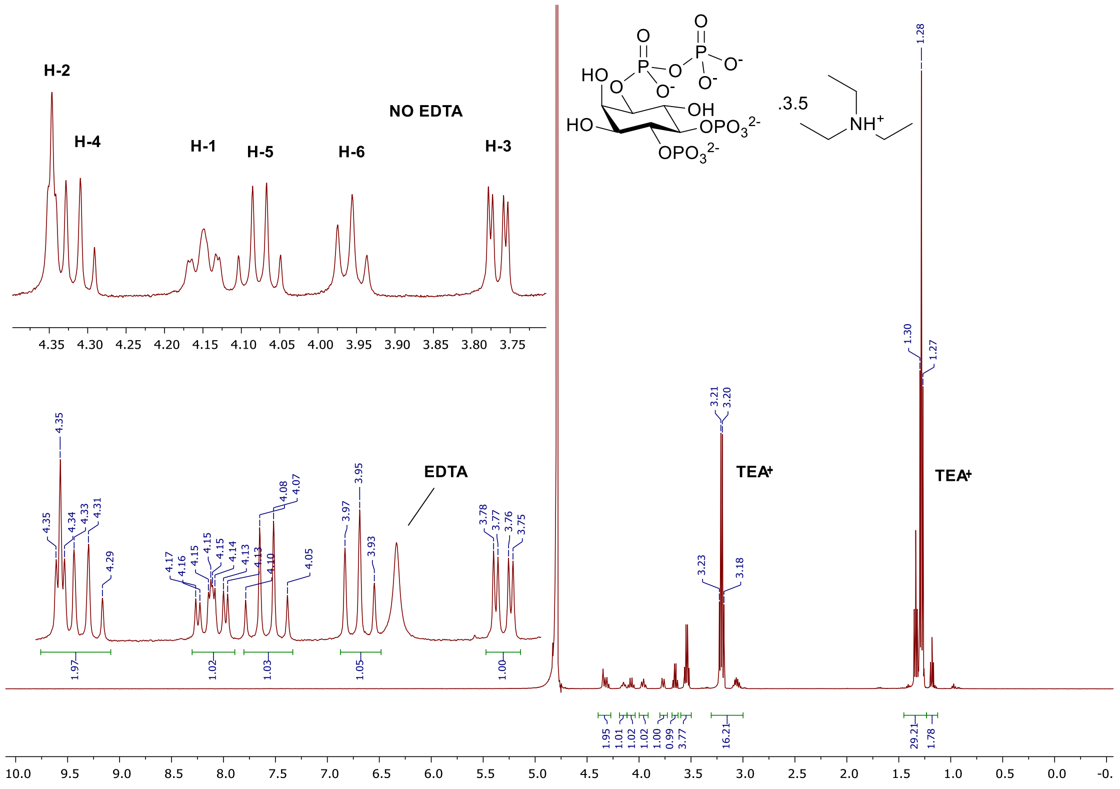

Note that adding a trace of EDTA gives a considerably sharper ^{1}H NMR spectrum. Some up-field signals around 1.3 and 3.2 ppm originate from trace alkylamine impurities in the trimethylamine used to make TEAB buffer for anion-exchange chromatography.

1-PP-Ins(4,5)P2 (1) ^{1}H-^{1}H COSY NMR (500 MHz, D$_2$O)

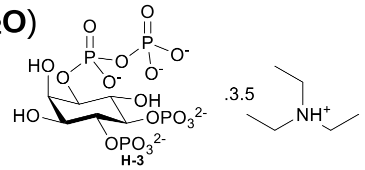

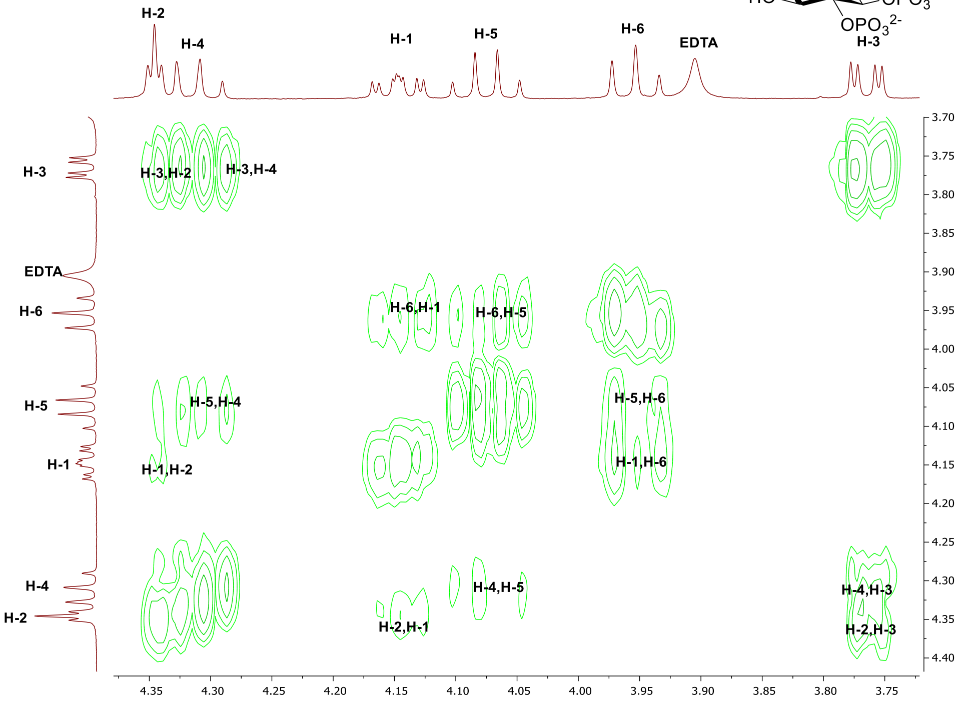

1-PP-Ins(4,5)P2 (1) ^{13}C NMR (100 MHz, D$_2$O)

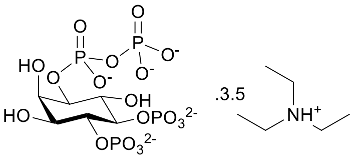

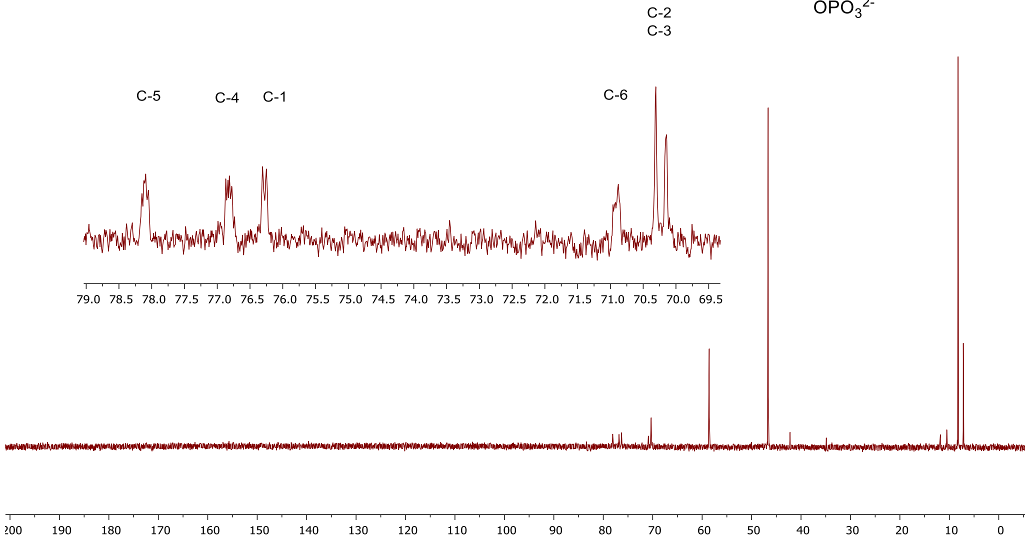

Some up-field peaks in the^{13}CNMR spectrum arise from trace impurities in the triethylamine used to make the TEAB buffer. They can be removed, if desired, by treatment with Chelex-100 resin (Na$^+$ form), but we have evidence that Na$^+$ salts of PP-InsPs may be less stable than their TEA$^+$ salt equivalents.

1-PP-Ins(4,5)P_2 (1) ^{31}P NMR (202 MHz, D_2O, ^{1}H-decoupled)

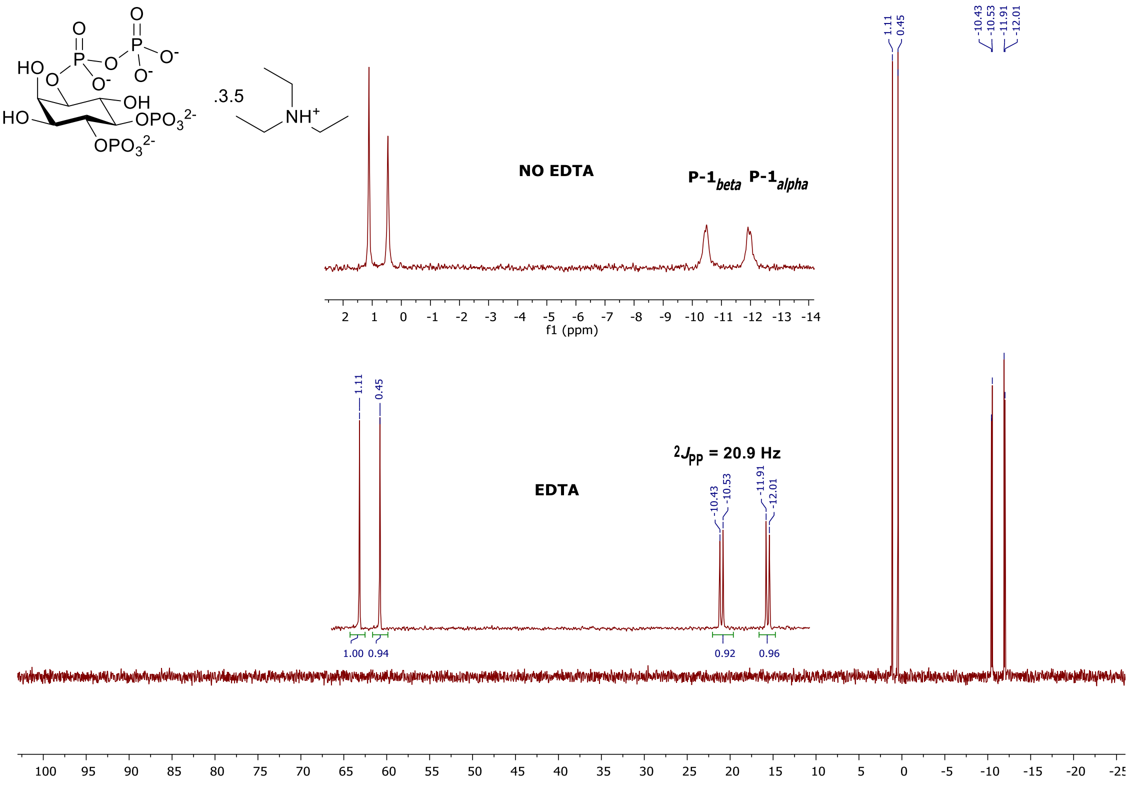

Adding a trace of EDTA gives a sharper ^{31}P NMR spectrum in D_2O, particularly for the peaks corresponding to the two phosphorus atoms ion the diphosphate group. This spectrum was unchanged after > 1 year in solution in D_2O at 4 °C.

1-PP-Ins(4,5)P_2 (1) ^{31}P NMR (202 MHz, D_2O, ^{1}H-coupled)

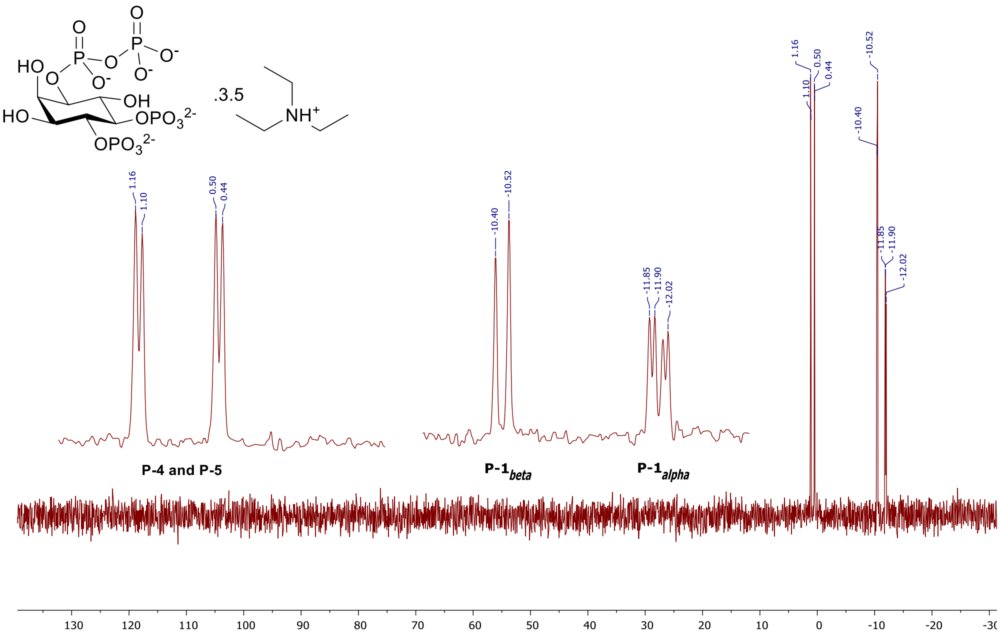

References

1. G. Jones, P. Willett, R. C. Glen, A. R. Leach and R. Taylor, *J. Mol. Biol.*, 1997, **267**, 727-748.
2. I. Bosanac, J. R. Alattia, T. K. Mal, J. Chan, S. Talarico, F. K. Tong, K. I. Tong, F. Yoshikawa, T. Furuichi, M. Iwai, T. Michikawa, K. Mikoshiba and M. Ikura, *Nature*, 2002, **420**, 696-700.
3. M. L. Verdonk, G. Chessari, J. C. Cole, M. J. Hartshorn, C. W. Murray, J. W. M. Nissink, R. D. Taylor and R. Taylor, *J. Med. Chem.*, 2005, **48**, 6504-6515.
